# Toward a roadmap for addressing today's health dilemma–The 101-statement consensus report

**DOI:** 10.3389/fnut.2025.1676080

**Published:** 2025-12-04

**Authors:** Katharina C. Wirnitzer, Mohamad Motevalli, Derrick R. Tanous, Clemens Drenowatz, Maximilian Moser, Holger Cramer, Thomas Rosemann, Karl-Heinz Wagner, Andreas Michalsen, Beat Knechtle, Zlatko Fras, Merel Ritskes-Hoitinga, Adilson Marques, Nataša Fidler Mis, Fatima C. Stanford, Christian Schubert, Nandu Goswami, Claus Leitzmann, Per Morten Fredriksen, Gerhard Ruedl, Doris Wilflingseder, Rodrigo A. Lima, Christian Kessler, Michael Jeitler, Naim A. Khan, Hassan Joulaei, Maryam Fatemi, Andrew Knight, Karl W. Kratky, Kara K. Palmer, Bernd Haditsch, Bostjan Jakse, Walter Kofler, Tomas Pfeiffer, Kathya Cordova-Pozo, Patrizia Tortella, Simon Straub, Heidi Lynch, Manuel Schätzer, Anupama Krishnan, Shahnaz Fathima A., Lukas Gatterer, Fabian Kriwan, Mittal Abhishek, Hemant Nandgaonkar, Shalaka Nandgaonkar, Abiola O. Adedara, Josep M. Haro, Corina Gericke, Gaby Neumann, Aysha Akhtar, Amir Rashidlamir, Madan Thangavelu, Gonza B. Ngoumou, Éva Perpék, Michael Klaper, Bhaswati Bhattacharya, Werner Kirschner, Kathelijne M. H. H. Bessems, Peter Jones, Gregory Peoples, Raul Bescos, Christina Duftner, Georg Seifert

**Affiliations:** 1 Department of Pediatric Oncology and Hematology, Otto-Heubner Centre for Paediatric and Adolescent Medicine (OHC), Charité – Universitätsmedizin Berlin, Berlin, Germany; 2 Charité Competence Center for Traditional and Integrative Medicine (CCCTIM), Charité – Universitätsmedizin Berlin, Berlin, Germany; 3 Department of Secondary Education, University College of Teacher Education Tyrol, Innsbruck, Austria; 4 Department of Sport Science, Leopold-Franzens University of Innsbruck, Innsbruck, Austria; 5 Division of Sport, Physical Activity and Health, University of Education Upper Austria, Linz, Austria; 6 Otto Loewi Research Center for Vascular Biology, Immunology, and Inflammation, Division of Physiology, Medical University Graz, Graz, Austria; 7 Human Research Institute, Weiz, Austria; 8 Institute for General Practice and Interprofessional Care, University Hospital Tübingen, Tübingen, Germany; 9 Robert Bosch Center for Integrative Medicine and Health, Bosch Health Campus, Stuttgart, Germany; 10 Institute of Primary Care, University of Zurich, Zürich, Switzerland; 11 Department of Nutritional Sciences, Faculty of Life Sciences, University of Vienna, Vienna, Austria; 12 Research Platform “Active Ageing”, University of Vienna, Vienna, Austria; 13 Institute of Social Medicine, Epidemiology and Health Economics, Charité - Universitätsmedizin Berlin, corporate member of Freie Universität Berlin and Humboldt-Universität zu Berlin, Berlin, Germany; 14 Department of Internal Medicine and Nature-Based Therapies, Immanuel Hospital Berlin, Berlin, Germany; 15 Medbase St. Gallen Am Vadianplatz, St. Gallen, Switzerland; 16 Division of Medicine, University Medical Centre Ljubljana, Ljubljana, Slovenia; 17 Medical Faculty, University of Ljubljana, Ljubljana, Slovenia; 18 Faculty of Veterinary Medicine, Institute for Risk Assessment Sciences (IRAS) Toxicology, Utrecht University, Utrecht, Netherlands; 19 Department of Clinical Medicine, Aarhus University, Aarhus, Denmark; 20 CIPER, Faculdade de Motricidade Humana, Universidade de Lisboa, Lisbon, Portugal; 21 Núcleo de Investigación en Ciencias del Movimiento, Universidad Arturo Prat, Iquique, Chile; 22 Independent Researcher, Ljubljana, Slovenia; 23 Department of Medicine-Division of Endocrinology- Neuroendocrine, Department of Pediatrics- Division of Endocrinology, Nutrition Obesity Research Center at Harvard (NORCH), MGH Weight Center, Harvard Medical School, Massachusetts General Hospital, Boston, MA, United States; 24 Department of Psychiatry, Psychotherapy, Psychosomatics and Medical Psychology, Medical University of Innsbruck, Innsbruck, Austria; 25 Center for Space and Aviation Health, Mohammed Bin Rashid University of Medicine and Health Sciences, Dubai, United Arab Emirates; 26 Institute of Nutrition, Justus Liebig University Giessen, Giessen, Germany; 27 Fakultet for Helse- og sosialvitenskap, Høgskolen i Innlandet, Lillehammer, Norway; 28 Ignaz Semmelweis Institute, Interuniversity Institute for Infection Research, Vetmeduni Vienna, Vienna, Austria; 29 Impact and Prevention of Mental Disorders, Parc Sanitari Sant Joan de Déu, Sant Boi de Llobregat, Spain; 30 CIBERSAM, Madrid, Spain; 31 Physiologie de la Nutrition & Toxicologie (NUTox), UMR 1231 INSERM/UB/AgroSup, Université de Bourgogne, Faculté des Sciences de la Vie, Dijon, France; 32 Health Policy Research Center, School of Medicine, Institute of Health, Shiraz University of Medical Sciences, Shiraz, Iran; 33 HIV/AIDS Research Center, Voluntary Counseling and Testing Center, Institute of Health, Shiraz University of Medical Sciences, Shiraz, Fars, Iran; 34 School of Environment and Science, Griffith University, Nathan, QLD, Australia; 35 Faculty of Physics, University of Vienna, Vienna, Austria; 36 School of Kinesiology, University of Michigan, Ann Arbor, MI, United States; 37 Preventive Medical Examination and Screening Centre, Austrian Health Insurance Fund ÖGK – Österreichische Gesundheitskasse, Graz, Austria; 38 Independent Consultant, Kranjska Gora, Slovenia; 39 Department Normal Physiology, I.M. Sechenov Moscow State Medical University (Sechenov University), Moscow, Russia; 40 Institute for Hygiene and Medical Microbiology, Medical University of Innsbruck, Innsbruck, Austria; 41 Institute for TCIM/CAM, Science and Research Department, Prague, Czechia; 42 Professional Chamber Sanator – the Union of Biotronicists of Josef Zezulka, Prague, Czechia; 43 Department of Research Methodology, Institute for Management Research, Radboud University, Nijmegen, Netherlands; 44 Department of Human and Social Sciences, University of Enna “Kore”, Cittadella Universitaria, Enna, Italy; 45 Department für Kinder- und Jugendheilkunde, Pädiatrie I, Gastroenterologie und Hepatologie, Universitätskliniken Innsbruck, Tirol Kliniken, Innsbruck, Austria; 46 Department of Kinesiology and Health Sciences, Point Loma Nazarene University, San Diego, CA, United States; 47 Special Institute for Preventive Cardiology and Nutrition (SIPCAN), Salzburg, Austria; 48 Department of Swasthavritta (Ayurvedic Branch of Preventive Medicine and Community Health), VPSV Ayurveda College Kottakkal, Kerala University of Health Sciences, Thrissur, Kerala, India; 49 Department of Orthopaedics and Trauma Surgery, University Hospital of St. Pölten, St. Pölten, Austria; 50 Department of Anaesthesia and Intensive Care, University Hospital of Innsbruck/Tirol Kliniken GmbH, Medical University Innsbruck, Innsbruck, Austria; 51 Amity Institute of Public Health, Amity University, Noida, Uttar Pradesh, India; 52 Department of Surgery, Vardhman Mahavir Medical College and Safdarjung Hospital, New Delhi, India; 53 Maharashtra University of Health Sciences (MUHS), Nashik, India; 54 Occupational Therapy Training School & Centre, Seth GS Medical College & KEM Hospital, Mumbai, India; 55 Occupational Therapy, Hands On Therapy Concepts Pvt. Ltd., Mumbai, India; 56 Department of Prevention, Care and Treatment (PCT), Institute of Human Virology Nigeria, Abuja, Nigeria; 57 Parc Sanitari Sant Joan de Déu, IRSJD, Universitat de Barcelona, CIBERSAM, IRSJD, Sant Boi de Llobregat, Barcelona, Spain; 58 Doctors Against Animal Experiments (DAAE), Bergisch Gladbach, Germany; 59 Center for Contemporary Sciences (CCS), Gaithersburg, MD, United States; 60 Department of Exercise Physiology, Faculty of Sport Sciences, Ferdowsi University of Mashhad (FUM), Mashhad, Iran; 61 Ayush Valley Foundation, Shoranur, Kerala, India; 62 Mind-Matter Unification Project, Cavendish Laboratory, Department of Physics, Theory of Condensed Matter Group, University of Cambridge, Cambridge, United Kingdom; 63 ELTE Centre for Social Sciences, Hungarian Academy of Sciences Centre of Excellence, Budapest, Hungary; 64 Moving Medicine Forward, Medical School Nutrition Education Initiative, St. Petersburg, FL, United States; 65 Department of Medicine, Weill Cornell Medical College, New York, NY, United States; 66 Center for Ayurveda Studies, Indic Academy, Hyderabad, Telangana, India; 67 Department of Health Promotion, Faculty of Health, Medicine and Life Sciences, NUTRIM Research Institute of Nutrition and Translational Research in Metabolism, Maastricht University, Maastricht, Netherlands; 68 Community Mental Health Teams, National Health Service (NHS), Wigan, United Kingdom; 69 Centre for Medical and Exercise Physiology/Graduate Medicine/School of Medicine, Faculty of Science, Medicine, and Health, University of Wollongong, Wollongong, NSW, Australia; 70 School of Health Professions, Faculty of Health, University of Plymouth, Plymouth, United Kingdom; 71 Department for Internal Medicine, Clinical Division of Internal Medicine II, Medical University of Innsbruck, Innsbruck, Austria; 72 Instituto de Tratamento do Câncer Infantil (ITACI), Departamento de Pediatria, Faculdade de Medicina, Universidade de São Paulo, São Paulo, SP, Brazil

**Keywords:** prevention, public health, chronic diseases, vegan, plant-based, physical activity, behavior, animal experiment

## Abstract

**Importance:**

In recent decades there has been an expansion in the quantity and quality of scientific findings and guidelines on different health topics to promote individual and public health status. Reports also indicate that there has been a simultaneous increase in the financial burden of disease, including trillions spent on healthcare resources by governments worldwide (predominantly in developing countries) to address health concerns. At the same time, personal health behavior is well-known as efficient and cost-free, holding four times the potential to prevent early death compared to health care. Despite this knowledge, data show that the increasing prevalence of unhealthy lifestyles and the associated chronic diseases, especially in Western societies, still need to be controlled. This circumstance exemplifies today‘s global health dilemma, which alarms the inadequacy of ongoing efforts to address the existing health concerns worldwide.

**Setting, insights, and observations:**

Three international, multidisciplinary, and inter-university events (two scientific conferences and one tertiary education symposium) were held in Austria (Innsbruck) between 2020 and 2022 to discourse, discuss, and debate these concerns. Two hundred eighty-four experts from 76 universities, organizations, and stakeholders spanning 31 nations and five continents participated in this international research and knowledge exchange to address today's global health dilemma. The latest scientific findings were discussed to develop practical strategies for improving lifestyle behavior and focus on the dual “Healthy Eating & Active Living” approach as a minimum recommendation for sustainable lifelong health and care. The expert panel debated crucial research priorities and future policies, identified gaps and untapped potential in basic health approaches that have been grossly neglected, and approved the evidence-based 101 consensus statements presented.

**Conclusions and relevance:**

These endeavors aim to develop novel and effective interventions that address the needs of individuals and communities and promote optimal health status.

## Highlights

- To address the global health dilemma of increasing non-communicable (chronic) diseases despite growing advances in health science and healthcare budgets, **it is the consensus of the panel of experts that the power of Lifestyle Medicine has the potential to significantly contribute to the “Prevention First” appeal. The dual approach to sustainable and lifelong health—“Healthy Eating & Active Living”—is the minimum recommendation inclusive of every individual for better public health**.
- To maximize health benefits for all, the permanent linkage of “Healthy Eating” is, at best, whole food plant-predominant, preferably vegetarian/vegan, and “Active Living” is, at best, daily and outdoors/in nature.
- This consensus statement aligns with the current trend towards de-medicalized and more holistic, personalized approaches to health and well-being, focusing on sustainable preventative policies.

## 1 Introduction, aims and methods

Despite groundbreaking accomplishments in science and technology over recent decades (1–4) health professionals currently face various challenges in addressing public health issues. These include financial interests, which can bias decision-making or priority-setting; difficulties in disseminating conclusive research to policy makers and the public in a timely and effective manner; and professional hubris, where overconfidence in expertise may hinder collaboration or the consideration of alternative approaches (5–9). Together, these factors have contributed to the increasing prevalence of non-communicable diseases (NCDs) and particularly their underlying risk factors (5–9). These challenges are primarily due to the broad and complex interface of health science disciplines and the contradictory viewpoints on health priorities. By bringing together experts and health professionals from diverse areas and communities through common ground, various foci could be openly linked in addressing current health issues of pressing concern.

The present paper reports the consensus and theme-specific results of three international, multidisciplinary, and cross-university events (10, 11) that hosted 284 internationally recognized experts from 76 universities, stakeholders, and organizations (including the WHO Regional Office for Europe) around the world (31 nations, five continents):

(1) “Health and Wellbeing: Addressing today's Global Paradox. Visioning an International Research and Knowledge Exchange”. 6. February 2020, 1-day hybrid conference, Innsbruck, Austria.
(2) “Improving Child and Adolescent Health for Better Public Health—Fiction or Within the Scope of Possibility? The perspective of a lifestyle-centered approach for Addressing Today's Global Health Paradox”. 10–11. November 2020, 2-day e-conference, Innsbruck, Austria.
(3) “The future in (y)our hands. Improved public health arises through better health of every individual. A tertiary education symposium about the future of human and planetary health”. 8. June 2022, 1-day hybrid conference, Innsbruck, Austria.

**Figure 1:**
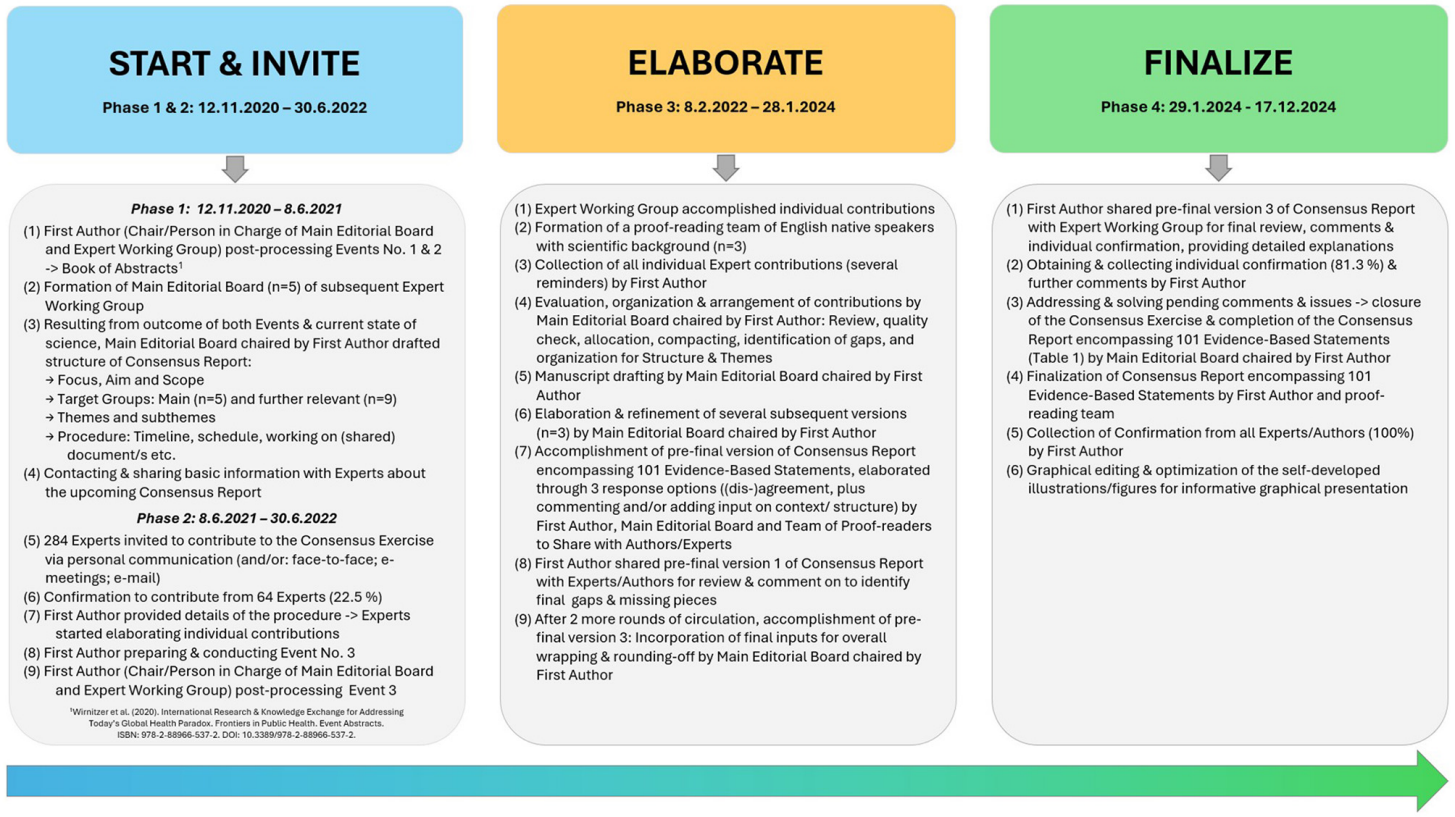
Flow Chart of Consensus Exercise Process using the ACCORD-method “Consensus Meeting” for health-related activities and research, chaired by the first author, 64 experts (22.54 %) from 62 entities, 20 nations, and five continents formed the working group (main editorial board: of physicians, public health experts, sport scientists: KCW, MM, DRT, CD, and WKo) in accordance with ACCORD (12). © Katharina C. Wirnitzer.

Through a consensus-generating approach (Figure 1, Supplementary Appendix S1), the experts (i) discussed the latest scientific findings including existing limitations in health approaches, (ii) identified gaps and untapped potentials in encouraging approaches that have been neglected, (iii) debated the necessity of specific and crucial research priorities, (iv) foresighted policies supporting novel and promising scientific and practical efforts, and (v) weighed on developing practical strategies to promote healthy lifestyle behaviors in everyday scenarios at different levels and settings with a specific emphasis on holistic and integrated health perspectives, particularly the preventative dual approach of “Healthy Eating & Active Living” (13–15) for sustainable, lifelong health and wellbeing.

The overarching aim of the congresses, within the context of an international research and knowledge exchange, was to establish direct statements to address today's global health dilemma—in the present context to be understood as a systemic paradox rather than a binary choice—which explores why growing advances in health science and increasing healthcare budgets do not adequately control the increasing prevalence of NCDs. This dilemma confronts policymakers and health care systems with competing, often undesirable trade-offs at the focal point of prevention, treatment, and long-term sustainability. Accordingly, it has been agreed that developing an evidence-based consensus statement outlining novel and effective intervention approaches to address the healthcare needs of individuals and communities is crucial for providing a scientific basis to promote an individual's optimal health status and improve the public health of nations worldwide. The aim was to focus on innovative interventions for improving the health outcomes of individuals and populations worldwide, which would reduce the social, ecological, and monetary burden of chronic diseases. As the German philosopher Arthur Schopenhauer (1788–1860) once famously exclaimed, “Health is not everything, but without health, everything is nothing.”

## 2 Background to the issue

### 2.1 Today's global health dilemma

Health is the natural aspiration of the human being from birth. Over the past few decades, the world has witnessed a shift in the primary focus of health concerns from infectious to chronic diseases, specifically NCDs (9, 16, 17). To control health problems, governments and international health organizations have established various policies over the past decades that have led to a general incline in healthcare budgets worldwide (18, 19) and are projected to cumulate to USD 15 trillion by 2050 (20). Accordingly, there has been rapid growth in the quantity and quality of health and medical investigations in recent decades (21, 22). Despite significant improvements in several areas of health sciences, such as sanitization/hygiene (23), neonatal health care practices (24), vaccination programs (25), and cancer screening (26), there are still gaps between the practical and applicable understanding of disease causes, the identification of biological markers of their presence and stage, and specific indicators influencing the effectiveness of potential remedies (27, 28). Therefore, the high-tech medicine of the 21st century has catalyzed today's global health dilemma, where despite significant advancements in health and healthcare, the world still experiences vast preventable deaths, high per-capita health burdens, and substantial financial costs.

In our technology-driven world, advanced and cutting-edge medicines are prioritized. It has been proposed that “omics” technologies, including proteomics, genomics, lipidomics, epigenomics, metabolomics, analysis of the microbiome, modern imaging methods, and physiological monitoring approaches, may once again play an important role, as they have already proven effective in fostering the implementation of precision medicine in daily practice (29–31). Based on this precision medical approach, specialists and physicians should ideally apply personalized treatment and consider the patient's unique physiology, microbial characteristics (viral, fungal, or bacterial properties), and the capacity to metabolize a particular medication and/or drug (31, 32). Contemporary advancements in science indicate the growing need to prioritize personalized approaches in medicine and expand our perspectives toward the person/patient as a whole organism. In 2022, NCDs accounted for 74% of all deaths globally (5); however, sustainably successful medicalized cures for preventable health conditions, particularly chronic health diseases with a more complex and multi-faceted etiology, such as obesity, diabetes type 2, cardiovascular diseases, and cancer, remain rare and elusive (33–36). While the theoretical aspects of modern medicine imply the necessity to apply three principles (diagnosis, therapy, and prognosis) to tackle the burden of diseases (37, 38), many current health approaches, specifically those to manage NCDs, are based on clear-cut diagnoses that often miss the underlying causes and subtler manifestations of illness (28, 39, 40). Such medical practice might lead to significant misinterpretation and may be—in some cases—significantly misleading.

The Continuum Concept, one of the most fundamental principles of physiology, biology, and disease, explains the status of an individual from optimal health to a hidden imbalance, progressing to severe dysfunction, ultimately leading to disease (41). A holistic and systematic approach to pathophysiology could shift models of disease in medicine away from simple associations rooted in empirical reductionism toward an appreciation of network-based models (42). An application of these systemic theoretical concepts in medicine can be seen, for instance, in the emerging field of psychoneuroimmunology, an interdisciplinary area of research exploring the complex interactions between psychological, neural, endocrine, and immune processes (43). While traditional medicine relies on specific regulatory types to handle treatment based on symptoms and the patient's general profile, it is also crucial to recognize cross-cultural factors in preventive and therapeutic approaches, particularly understanding shared and distinct pathological, physiological, and psychological features (44). It has been shown that personal behavior is the predominant determinant of health today, with four-fold the power to prevent premature death compared to healthcare (45, 46). Therefore, to promote overall health and wellness, it is essential to encourage behaviors that benefit the entire complex rather than isolating specific organs, tissues, cells, or structures. Unhealthy lifestyle behavior (e.g., physical inactivity, poor dietary habits, and substance abuse), along with overweight/obesity, is the primary mechanism of the pressing NCD challenge (5, 47, 48). To tackle these global health issues that predominantly track from childhood to old age (49, 50), it is crucial to address the pressing concerns identified by health organizations and experts worldwide (51, 52).

### 2.2 Health and the power of lifestyle

**Figure 2:**
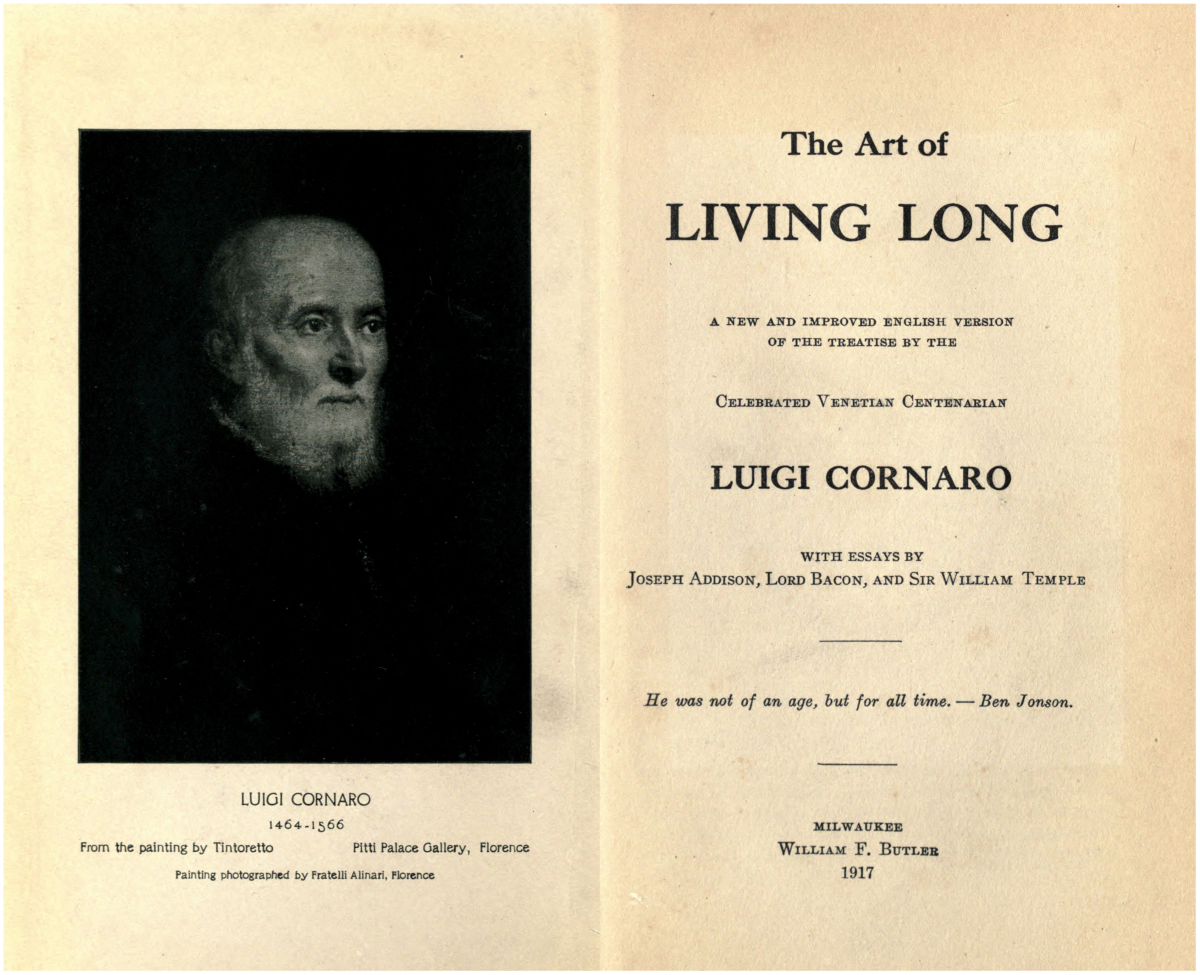
‘The Art of Living Long”. A new and improved English version of the Treatise by the celebrated Venetian Centenarian Luigi Cornaro (Butler, 1917; without page) (54). Photography of the painting by Tintoretto of Luigi Cornaro (Fratelli Alinari, undated). Digitalized by the Internet Archive (2007). Digital reproduction: California Digital Library, Public Domain.

The desire for a happy and long life (with as few years lived with disability as possible–especially at the end of one's lifespan) has been a fundamental human aspiration and traces back to the beginning of humankind (53). In 1550, however, Luigi Cornaro (an Italian humanist, 1475–1566; Figure 2) reported for the first time that the human lifespan can be extended by making lifestyle modifications (54, 55). This idea is consistent with the current body of science, which indicates that genetics account for 20%−25% of an individual's lifespan, while the remaining 75%−80% is determined by various factors but principally lifestyle (56–58).

**Figure 3:**
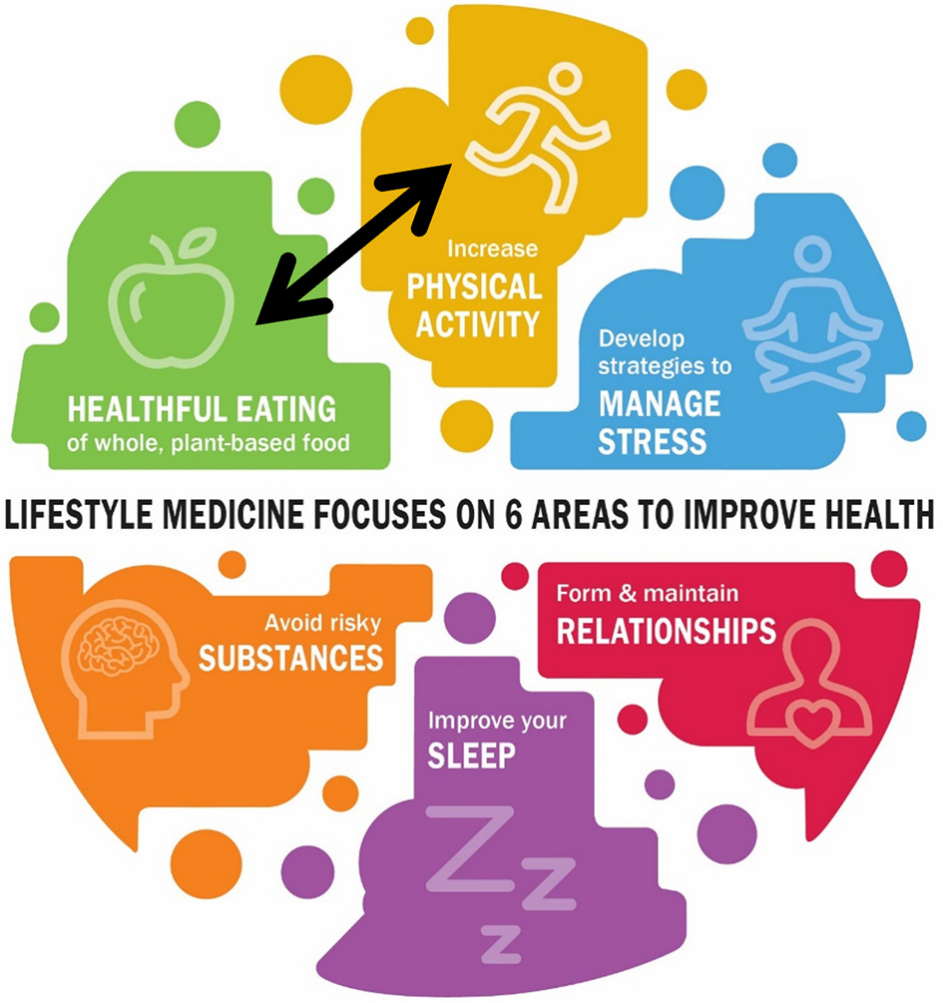
The six areas of lifestyle medicine – healthful eating, physical activity, stress, relationships, sleep, and substances – are complex and interconnected factors that substantially impact an individual's overall health and well-being. These areas, recommended briefly as healthy eating, keeping moving, sleeping well, avoiding substances, staying calm, and loving people (60), are influenced by various personal, environmental, and social factors contributing to their intricate nature. “Healthy Eating & Active Living” forms the minimum recommendation to start one's engagement toward better health. Adapted from (60) with permission from American College of Lifestyle Medicine (ACLM). Graphic modification: © Katharina C. Wirnitzer.

Today, the lifestyle medicine concept is a concrete, evidence-based, and comprehensive approach to preventing, treating, and even reversing various diseases by implementing health-promoting behaviors and replacing poor/unhealthy ones that are disadvantageous to health in daily routines. According to the American College of Lifestyle Medicine (ACLM), there are six pillars of lifestyle, including a whole-food, plant-predominant eating pattern, physical activity (PA), stress management, positive social relationships, restorative sleep, and the avoidance of risky substances (59, 60) (Figure 3). These six areas form the foundation of the recently designed “Lifestyle Medicine Curriculum,” which has been integrated into medical education (61). Lifestyle medicine aims to reduce dependence on medical treatments such as pharmaceutical therapy (prescribed medication) or surgical strategies by strongly emphasizing health-related behaviors. Instead, Lifestyle Medicine taps the potential of healthy dietary habits, regular PA, stress management techniques, positive relationship reinforcement (solid social support), improving sleep quality, and restricting smoking and alcohol intake (59, 60).

In line with and as an extension of lifestyle medicine, evidence suggests that Traditional, Complementary, and Integrative Medicine (TCIM) may significantly manage various health problems (62). TCIM is typically used alongside modern evidence-based medical approaches and is classified into five major categories: “whole medical systems,” “mind-body techniques,” “biologically based practices,” “manipulative and body-based therapies,” and “energy therapies” (63). TCIM (64), which is closely tied to cultural aspects, has received endorsement from the European Union (EU) (65), WHO (66), and other intergovernmental organizations (67–69). Considering that the cost-effectiveness of TCIM-based treatments is approved by the European Parliament (70) and may reduce concerns associated with the rising cost of modern medical practices, such health management approaches should be considered in future healthcare efforts worldwide.

### 2.3 “Healthy Eating & Active Living”

As one key component of a healthy lifestyle, a person's diet has the power of medicine and has thus been applied in treating various health conditions beyond preventative effects (71–76). Dietary patterns (e.g., type of diet: omnivorous, vegetarian) have always been influenced by food preferences and availabilities of geographical, sociocultural, economic, and religious factors (77–79), which have uncovered the complexity of defining a healthy diet (80–86). Available dietary guidelines have consistently emphasized replacing unhealthy food options with healthy ones (e.g., fruits, vegetables, whole grains, beans, legumes and pulses, nuts, and seeds) (81–91). While there are wide discrepancies in defining a healthy diet (71, 78), it has been well-documented that a whole-food plant-predominant (WFPP) diet (most significantly through a vegetarian or vegan pattern) is a general description for a healthy dietary approach (14, 84, 92–97). Notwithstanding the clarifications provided by scientific evidence, several misconceptions persist regarding the adverse health effects of transitioning to a vegetarian diet, especially a vegan one. However, such beliefs may lack scientific rigor, as they often result from a limited understanding of how to choose a diverse array of plant foods as substitutes for animal-sourced foods, as well as the potential for unbalanced and poorly planned dietary regimens, which can occur in any diet (93, 98). As so-called critical nutrients of the vegan diet (e.g., iron, iodine, calcium, vitamin D, vitamin B12) are not more problematic than any other diet type, it appears that vegans are at no exceptional risk for deficiency with a well-composed, wholesome diet appropriately supplemented by vitamin B12 (93). Hence, regardless of diet type, nutritional inadequacy usually emerges from other factors, primarily based on individually misapplied dietary behaviors (99–101).

A well-planned healthy diet has been documented as the most simple, safe, accessible, and affordable way to prevent and treat various diseases such as cardiovascular disease, type 2 diabetes, and the associated risk factors, including hypertension, hyperglycemia, and obesity (14, 88–90, 102, 103). Conversely, poor or unhealthy diets significantly contribute to global mortality, with dietary risks identified as one of the leading risk factors for death and disability, accounting for 20% of the cases worldwide (47, 104). For instance, data show that cardiovascular diseases, the world's leading causes of death over the past two decades (104, 105), are reversible by diet alone but only when adhering to a healthy, WFPP (especially vegan) diet (106–108). Healthcare approaches are highly connected to medical education. Nowadays, medical education and training seem to be primarily disease-centered, based on the specific knowledge of diseases and medical approaches. At the same time, health promotion is almost absent in curricular-determined learning and training content, which is a reflection of out-of-date education and practice (109–112). Results from a systematic review show that medical students lack the competence to provide high-quality, adequate nutrition and lifestyle care (113). Such circumstances have directed healthcare systems toward being more reactive than proactive, prioritizing treatment of illnesses over prevention, ultimately resulting in massive expenses to medically intervene (114–116). However, since 2019, policymakers in the EU emphasized the urgent need for more significant and more pressing efforts toward a shift to the prevention of ill health and disease and pose health promotion as a key pillar in ensuring future public health across nations (114). Accordingly, there has been an emphasis on lifestyle-based approaches that need to be included in the current healthcare system and the associated medication-based, disease-centered programs (117–119). The American Medical Association (AMA) consistently advocated for implementing plant-predominant meal options in hospitals and medical facilities (120). This initiative extends beyond merely improving the health of patients, staff, and visitors while encouraging the elimination of animal products from the menu (120). However, today's health dilemma has also raised concerns among primary care physicians and pediatricians regarding vegan/vegetarian nutrition, primarily due to a lack of detailed academic education on critical topics. The lack of compulsory education covering the depth of vegetarian dietary patterns and health makes it challenging for medical doctors to effectively support, recommend, or interact with individuals following non-omnivorous dietary patterns (98, 119, 121).

The history of reporting on plant-predominant diets dates back over 2,500 years, when Pythagoras introduced the philosophy of vegetarianism based on his observations (98). This nature-based philosophy, which generally prevailed until the 19th century, often recommended diets predominantly composed of plant foods to maintain health and wellbeing as well as alleviate and heal disorders and diseases (98, 122). However, during the past century (especially after World War II), profound changes in dietary habits have occurred worldwide: mainly including changes from (i) meals to snacks, (ii) eating at home to eating out, (iii) eating fruits and vegetables (plant-rich diets) to eating more animal foods; and (iv) eating intact, fresh, and organic foods to processed and industrialized products high in sugar, salt, (saturated) fats, and preservatives (78, 95, 123, 124). These changes, which have been associated with the increasing prevalence of NCDs and the risk factors for global mortality (5, 6, 125, 126), have led to the emergence of a vast number of research efforts since 1978, investigating and displaying evidence on the effectiveness of different types of diets, especially plant-predominant diets on sustainable health promotion (92, 100–103, 127–134) and longevity (108, 135, 136). Blue zones (specific locations around the world with populations living significantly longer and in good physical condition) are natural examples of the relationship between plant-predominant diets and healthy longevity (137, 138). Regarding the sustainability aspect of dietary maintenance, the EAT-Lancet Commission on healthy diets from sustainable food systems is considered a giant step (139). Even more recently, the European Commission (EC) has advocated Food 2030 as a framework for research and innovation policies, aiming to facilitate the transition toward sustainable, nutritious, and inclusive food systems (140). Furthermore, the latest Intergovernmental Panel on Climate Change (IPCC) summary report for policymakers highlights that shifting to a plant-predominant diet across all socio-ecological levels holds the most significant potential to protect individual and global health, promote human sustainability, including mitigating infectious diseases, epidemics, pandemics, and resource depletion along with increasing active mobility and other beneficial actions for health (141–143).

In parallel with the occurrence of dietary changes over the past century, the advancements and implications of technology in the daily routines of human beings have developed a new health concern worldwide called “sedentary lifestyle” (144). The effect of PA, sports, and exercise on NCDs has been widely studied over recent decades, and it has been proven that physical exercise is a versatile and effective treatment of NCDs, thus considered as medicine (145–151). As a crucial part of a healthy lifestyle nowadays, PA, sports, and exercise are critical factors in maintaining physical, physiological, and mental health and wellbeing across the life course, which is influenced by interpersonal and environmental factors (152–154). Rather than having specific and direct benefits for health, natural settings for performing PA, sports, and exercise promote health-enhancing behavior (155–160). There is scientific agreement that properly planned and regularly carried out PA (at best daily and outdoors/in nature) seems to act as a preventive measure, delaying or avoiding the onset of certain diseases, and as an effective treatment for subjects already affected by NCDs by increasing the quality of life, slowing down the progression of illness, and/or eradicating it (146–151, 158, 159). According to the general health guidelines of the World Health Organization (WHO) (145, 154), adults are recommended to comply with at least 150 min of moderate-intensity PA per week distributed over at least 3 days (at best, a minimum of 20 min daily) (161–163), while children and adolescents (aged 5–17 years) are advised to engage in at least 60 min of moderate-to-vigorous intensity PA daily, with mostly continuous activities involving large muscle groups.

Research advancements in health behavior have revealed the cumulative health benefits of PA and diet when applied simultaneously. Specifically, a healthy diet and regular PA, each as a single, 1-dimensional strategy, not only play independent roles in preventing and treating NCDs and associated risk factors *per se*, but their combination brings about additional synergistic benefits (164–166). This fact suggests the adoption and adherence of a holistic approach to health behavior by integrating all areas of lifestyle medicine, with a particular focus on the two main pillars of sustainable health–“Healthy Eating & Active Living”–forms the minimum recommendation to start one's engagement toward better health behaviors, habits, and patterns (Figure 3) (13–15, 165–172). This dual approach to sustainable and lifelong health is in line with contemporary views on human physiology and health (173–175), which requires a systemic perspective that integrates multimodal data from various entities to create a network functioning in health and disease and to explore its dynamic responses to disturbances (176). These integrative and multidimensional interactions shape the system's behavior and must be considered comprehensively over time, space, and the context of appearance (177, 178). Therefore, a holistic, de-medicalized, and systemic approach to health could move models of preventive and therapeutic strategies from simple associations rooted in empirical reductionism toward the appreciation of network-based models (29, 45, 179–182).

### 2.4 Environmental and ethical concerns in health promotion efforts

The benefits of healthy lifestyle behavior are not limited to the promotion of individual health or the public health of nations. Each component of a healthy lifestyle (e.g., consumption of plant foods, regular engagement in PA, sports, and exercise, etc.) considers ethical, social, and ecological aspects directly and indirectly (183–185). The United Nations' 17 Sustainable Development Goals (SDGs) mainly aim to end (#1) poverty and (#2) hunger globally (186). To do so, however, the most critical steps are achieving (#3) good health and wellbeing simultaneously with (#4) quality education (186), especially for women (187). From a broader perspective, personal health behavior is connected to planetary health and can significantly contribute to preserving and maintaining human development (185, 188). In parallel, ecosystem changes, particularly climate changes (primarily originating from human behavior over the past century), pose a significant threat to human health (188–191). In addition to increasing the likelihood of natural disasters (192), anthropogenic climate change has gradually led to environmental alterations, such as air pollution, loss of biodiversity, resource depletion, more frequent and heightened heatwaves, deforestation, and exacerbating water shortages, which may result in a wide range of health concerns, including instability and insecurity of resources, particularly food insecurity (180, 187, 188). However, the implementation of holistic solutions that consider synergies and trade-offs to target the United Nations' SDGs (181) and simultaneously address health and climate change challenges is restricted by the lack of action by decision-makers at both the national and global levels (189, 193–196). The WHO estimates that between 2030 and 2050, climate change will cause an additional 250,000 deaths globally each year due to malnutrition, infectious diseases, and heat stress (197). Children are among the most vulnerable populations, as recent reports from UNICEF indicate that about one billion children worldwide are at high risk of the impacts associated with climate change (198).

With a new and future-oriented holistic and de-medicalized lifestyle approach, critical concerns about animal testing in the pursuit of scientific progress could be addressed and tackled. Since the Medieval era, animal experimentation has driven medical research and justified medical progress. It is still considered the gold standard to advance knowledge in various fields, such as life science, medicine, agriculture, or product safety (199, 200). In 2012, almost half of the National Institute of Health's funding was allocated to testing that involved animal use (201). Today, however, there is ongoing questioning about the contemporaneity of vivisection and the utility and ethical acceptability of invasive animal research. Irrespective of ethical concerns regarding the justifiability of animal experiments in research activities, animal experimentation may not align with precision medicine approaches, which focus on the genetic diversity of human populations and inter-individual differences based on non-hereditary alterations during the life course (29, 30). This fact is evident, for example, in many human-specific diseases such as Alzheimer's, Parkinson's, multiple sclerosis, rheumatoid arthritis, type 1 diabetes, cystic fibrosis, and hemophilia A, which do not appear in animals (202, 203).

Furthermore, evidence shows that most (up to 95%) pharmaceutical drugs found effective and safe in animal studies fail in subsequent clinical phases in human populations, mainly due to insufficient efficacy, toxicity, and the emergence of unacceptable adverse drug reactions, raising significant concerns for ethical and economic reasons (204–211). This failure is predominantly attributed to the limitations of animal models in accurately representing human physiology during preclinical evaluations (211). It should also be considered that even if a drug is approved, safe use on humans is not guaranteed, as about one-third of the approved drugs are later withdrawn from sale or have warnings issued due to serious side effects (212). In September 2021, the European Parliament passed a resolution with 99% support to phase out animal testing across member countries, focusing on plans and actions to accelerate the transition to innovation without using animals in research, regulatory testing, and education (213). The EC is currently preparing a roadmap for phasing out animal studies for chemical safety testing in 15 legislative domains, which will be published at the beginning of 2026 (214). The EC responds to the European Parliament and the European Citizen Initiative to commit to a Europe without animal testing, collecting more than 1.2 million signatures from citizens (215). As a preparation, several transdisciplinary recommendations on how to become successful have already been published (216). Similarly, in December 2022, U.S. President Biden signed the FDA Modernization Act 2.0 into law, with strong bipartisan support, to speed up the discovery process, lower the costs of new life-saving treatments, and reduce animal testing (217). As a follow-up, an FDA roadmap has been published, stating that in 3–5 years time human-relevant testing methods (non-animal New Approach Methods) will be the default for safety/toxicity testing instead of animal studies (218). The National Institutes of Health in the US have followed suit in prioritizing the development and use of human-relevant testing methods by stating that projects only encompassing animal studies will no longer be funded (219).

### 2.5 Health education

Health education is not solely limited to the medical society, including healthcare practitioners. Children, adolescents, and young adults are widely recognized as vulnerable populations regarding health behavior, and promoting their health is crucial for their growth and future human development (220–223). Given that young people spend most of their daily activities in school as well as other educational contexts throughout a significant portion of their lifetime (224), it is essential to understand that schools and universities are vital settings for delivering health-related knowledge and developing competencies through adequate and effective education and training (220–222). Moving to a higher educational level may also bring about challenges regarding significant life changes in peer social circles and social pressures as well as educational environments, assignments, and facilities, thus permitting quick-fix, unhealthy lifestyle choices (225–227). However, the opportunity to provide education and training on healthy lifestyle behavior seamlessly linked from kindergarten, primary, and secondary school to college and university remains available according to national curricula, and it is yet to be fulfilled (221, 228). The WHO recommendations on health education emphasize that school and educational policies should support the adoption and maintenance of healthy lifestyle behaviors as a requirement for improving public health (220) and thus created the standard of “Health promoting schools” (WHO's Global School Health Initiative), launched in 1995 (229). Kindergartens, schools, and universities are living, working, and, at best, health-promoting environments that provide an optimal educational context for shaping lifelong healthy lifestyle behavior (230–232). Likewise, through educational efforts, the public is entitled to know about the controversy surrounding disease definitions and evaluations and the self-limiting and relatively benign natural course of many health conditions (233). Overall compulsory education is the optimum period (8–15 years in Europe) (234) in the life cycle where young people can be educated, trained, and empowered for healthy lifestyle behavior (234–236).

Nowadays, based on the overarching educational goal of “health promotion” as a curricular state mandate of many Western nations, it is therefore relevant that all compulsory subjects are competence-oriented and applied from the primary level to secondary level II (237–239). However, the central issue in translating conclusive, health-related scientific evidence into robust behaviors in adults and older adults is the reduced ability to learn new healthy behaviors after childhood and adolescence (240–243). Along with this, aptly formulated ancient wisdom was proclaimed by Confucius, a Chinese philosopher, politician, and educator: “If your plan is for 1 year, plant rice. If your plan is for 10 years, plant trees. If your plan is for 100 years, educate children.” Typically, compared to adults, health conditions may have a higher value in infancy, childhood, adolescence, and young adulthood, during which an individual faces several changes and transition phases. Outside of young people's family/guardianship and upbringing, it is generally recognized that early-stage education is critical for establishing long-term, health-related knowledge, skills, and competencies to achieve health policies. Developing a sustainable and lifelong health-related action readiness is essential, as well as the willingness to act wholesome; as the German writer Johann Wolfgang von Goethe (1749–1832) stated: “Knowing is not enough; we must apply. Willing is not enough; we must do.”

## 3 101 evidence-based consensus statements and research priorities

Table 1 presents a condensed overview of the 101 evidence-based consensus statements and research priorities along with respective rating the quality-level of evidence (Quality Rating Scheme for Studies and Other Evidence, modified from Oxford Center for Evidence-Based Medicine, with ratings from 1: properly powered and conducted randomized clinical trial/systematic review with meta-analysis; to 5: opinion of respected authorities; case reports).

**Table 1:** Condensed overview of the 101 evidence-based consensus statements and research priorities along with respective rating the quality-level of evidence (Quality Rating Scheme for Studies and Other Evidence, modified from Oxford Center for Evidence-Based Medicine: 1 = properly powered and conducted RCT/systematic review with meta-analysis; 2 = lesser-quality RCT or high-quality cohort study; 3 = case-control or retrospective cohort study; 4 = case series or poor-quality cohort/case-control studies; 5 = opinion of respected authorities or case reports).

**101 Evidence-based consensus statements and research priorities**		**Level of evidence**	**Suppl. sources**
***Theme 1. General Aspects of Health Behavior*** **(n** = **22)**			
1	Addressing the detrimental effects of chronic unhealthy behaviors throughout one's lifespan requires treatment by healthy lifestyle behavior and merely relying on short-term therapeutic interventions such as pharmacotherapy or surgical techniques is neither adequate nor permissible	5	(59, 60)
2	The definition of health is fundamentally rooted in holistic well-being, which underscores the importance of directing preventive and promotive health approaches toward this overarching goal	5	(168, 175)
3	It is imperative to consider personalized indicators of health behavior, not only in the diagnosis, prognosis, and treatment of specific health conditions but also in tailoring effective preventive and promotive health strategies. Personalized health behavior programs have the potential to yield superior health outcomes while also proving to be a cost-effective measure in terms of saving time, money, and energy for all parties involved	5	(30, 32)
4	By providing individuals with the tools and resources to develop healthy habits that are specific and relevant to their own lives, they are more likely to feel empowered by competence-oriented education and training and motivated to make positive changes, which goes together with action-readiness and the willingness to act internally. Personalizing motivation can involve goal setting, social support, self-monitoring, and feedback	5	(244, 245)
5	In clinical settings, addressing the early stages of NCDs necessitates the implementation of healthy lifestyle interventions for a specific duration rather than prioritizing pharmaceutical or surgical interventions as an initial approach	5	(16, 117)
6	Sustainable healthy behavior not only promotes individual well-being but aligns environmental and social principles and contributes to the stabilization and maintenance of planetary health	5	(194, 246)
7	The dynamic and constantly evolving nature of individual and public life exerts significant influence on health behavior, creating a challenging environment that requires timely adaptation of health-related approaches to keep pace with the latest technological and societal advancements. As such, the development and promotion of health strategies and guidelines (based on the latest scientific evidence) must be emphasized to align with the evolving lifestyle and behavioral habits throughout an individual's life	5	(173, 184)
8	Contemporary health promotion strategies must incorporate innovative and technologically upgraded methods to devise efficacious solutions to address health issues predominantly driven by modern technological developments (e.g., implementing exergaming to counter the sedentary lifestyle induced by machinery and the digital age)	5	(28, 247)
9	Both the domestic (mainly family) and the professional (e.g., kindergarten/school, college/university, job) environments where individuals' grow and learn/work play significant roles in developing and shaping health behavior as they can directly impact an individual's access, availability, and exposure to health-promoting or health-compromising factors	1	(155, 185)
10	Adopting a new healthy lifestyle behavior in adulthood and older adulthood is challenging; therefore, starting early in life is the foremost opportunity for establishing and expanding healthy behaviors into the lifestyle. Ideally, this should begin already during early childhood (i.e., in the home environment as well as in kindergarten) and solidify throughout childhood, adolescence, and emerging adulthood	1	(50, 226)
11	Compared to other populations, vulnerable groups such as infants, children and adolescents, elderly individuals, and those with a background of NCDs necessitate a greater emphasis on de-medicalized, holistic, preventive, and promotive health approaches	5	(16, 248)
12	Circadian rhythm cycles play a crucial role in evaluating and improving various aspects of health. If this rhythm cannot be adjusted based on health programs (e.g., in shift workers), the work schedule should be considered in health promotion intervention plans	5	(177, 249)
13	Providing flexibility in health behavior programs can incentivize individuals who still need to adopt a holistic, healthy lifestyle to participate in preventive and promotive health approaches.	5	(175, 177)
14	Regular assessment and seamless monitoring of health status, behaviors, and habits, including body composition, physiological parameters (such as clinical biomarkers and underlying mechanisms), and mental and psychological well-being, is a potent strategy to indirectly motivate the target populations in different educational and occupational settings toward enhancing their commitment and involvement in health promotion plans and measures	5	(177, 250)
15	To ensure more effective and practical health-related research, funding agencies should adopt purposeful review approaches by prioritizing and integrating the study design and methodological aspects of research activities such as randomized controlled trials, cross-sectional and cohort studies, and systematic reviews. This can also help reduce the production of pseudoscientific claims	5	(37, 251)
16	Advocating public health policies that facilitate healthy lifestyle choices is crucial for better or worse health, as the optimal default should be the opportunity for any individual to lead a healthy lifestyle with ease	5	(237, 252)
17	Private and public sector companies should prioritize promoting healthy lifestyles among their employees, including encouraging participation in worksite health and well-being measures and programs. Such initiatives can benefit individual and public health and employee efficiency at work, resulting in a return on fiscal health investments in human resources for the economic business and public sector	5	(179, 253)
18	Enhancing access to healthy lifestyle opportunities and programs in diverse educational and community settings and contexts is essential for promoting healthy habits and behaviors among low-income populations and practical decisions considering hands-on solutions should be made to achieve this goal	5	(239, 254)
19	Given the strong influence of environmental factors on health behaviors, it is essential for future research to prioritize the development and implementation of local and community-based interventions and study designs that yield representative findings that are synthesized with local, federal, national, and international approaches, guidelines, and recommendations to enable increases in successes of public and global health agendas and policies	5	(253, 254)
20	Initiating evidence-based policies and advocating for direct and indirect legislation that promotes holistic and de-medicalized health behavior is essential for easy implementation in everyday life. Innovative approaches are necessary to increase opportunities for sustainable and lifelong adherence to healthy behaviors and habits	5	(30, 250)
21	The assessment and comparison of health behavior policies across nations should evaluate respective priorities, deficiencies, and efficacy to culminate a cohesive presentation of these findings with global implications, serving as up-to-date guidelines and recommendations	5	(186, 195)
22	As a feasible, cost-effective approach for individuals of all ages, “Healthy Eating & Active Living” is a practical and sustainable method to attain good health, in line with the European Commission‘s “prevention first” appeal. This dual approach, as a minimum recommendation to sustainable and lifelong health, is most promising because it is not only beneficial for individual health and has the potential to shape better public health of nations for future generations to come but is also advantageous for ethical, social, ecological, and economic reasons	1	(167, 168)
***Theme 2. Healthy Eating (n** = **19)***			
23	Adhering to a healthy diet can be more effective (and without adverse effects) than medication therapy for NCDs	1	(71, 255)
24	The dietary characteristics of a healthy lifestyle are based on the quantity and quality of one's dietary pattern, including food choices and the timing and composition of meals, which is generally translatable to two fundamental principles: (i) quantity: avoiding a constant positive energy balance, ensuring that energy intake does not usually exceed energy requirements; and (ii) quality: increasing the consumption of fresh and whole plant foods, such as vegetables, fruits, beans, legumes and pulses, grains, tubers, nuts, seeds, and herbs, while also maintaining proper hydration (preferably by water)	1	(90, 91)
25	There is consensus that well-planned WFPP, preferably vegetarian and vegan, diets (i.e., care with planning, appropriate composition, and preparation) are (i) nutritionally adequate and provide sufficient calories that match dietary guidelines and meet current recommended intakes, (ii) healthy and provide unique benefits for the prevention and treatment of a variety of diseases, and (iii) appropriate at all stages of the life cycle and for athletes. Vegans must ensure an adequate supply of vitamin B_12_ through reliable sources (i.e., fortified foods and supplements)	1	(106, 111)
26	Adopting a healthy eating approach, specifically by following a WFPP diet (preferably vegetarian or vegan), can exert an auspicious impact on gene expression by reducing the expression of unfavorable genes while promoting the expression of advantageous genes, thereby passing substantial benefits to future generations	1	(111, 256)
27	Organic, plant-predominant, whole-food items may provide additional health benefits, such as reducing exposure to harmful substances like synthetic pesticides and offering higher levels of certain nutrients	5	(93, 111)
28	Compared to processed and preserved food options (except fermented foods like sauerkraut, miso, etc.), fresh food choices can provide higher nutrient density, better taste, lower exposure to harmful substances, and are more environmentally compatible by reducing the carbon footprint associated with transportation, packaging, and storage	5	(83, 93)
29	Preparing and cooking plant-predominant foods with gentle methods such as raw-food methods, steaming, baking, and fermenting can maintain their health benefits by preserving nutrients and promoting the bioavailability of phytochemicals	5	(83, 93)
30	Combining a healthy diet and the communal practice of sharing mealtimes with family, friends, and classmates at school can benefit physical and psychological well-being by promoting social connection and support	5	(220, 224)
31	Shopping habits are a crucial determinant of dietary patterns, as the availability and accessibility of healthy foods can significantly impact food choices and, ultimately, overall diet quality and health outcomes. In this regard, vegan and vegetarian food options should be more affordably priced than animal products, which can encourage more people to choose healthier foods	5	(77, 212)
32	Given the well-established, bidirectional relationship between mood swings and food consumption regarding quantity and taste preferences, it is essential to consider mindful eating as a crucial strategy when developing a healthy dietary plan, particularly for individuals with abnormal eating patterns	5	(167, 257)
33	Poor socioeconomic status, time limitations, and cultural norms surrounding food provisions can, independently or collectively, significantly impact food choices and ultimately lead to poor diet quality and adverse health outcomes	1	(77, 78)
34	Overcoming the hindrance of motivational inadequacy, sociocultural obligations, and socio-economic inequities to adhere to productive, healthy dietary changes requires a multidimensional approach at the interface of a variety of disciplines, including personalized goal setting, social support, and tailored nutrition education to empower individuals for informed nutrition choices by evidence-based knowledge, skills, and competencies enabling them to choose their dietary options wisely	5	(93, 96)
35	A positive and congenial atmosphere while eating is essential for promoting optimal digestion, maximizing nutrient absorption, and improving overall physical and mental health	5	(71, 85)
36	Considering food technology, policies promoting healthier attributes in the food industry's products (such as reducing portion sizes and utilizing less refined ingredients, sugar, saturated fats, and salt) can effectively promote healthier dietary choices	5	(81, 97)
37	To facilitate the adoption of healthy food options, it is essential to introduce and provide individuals with opportunities to sample nutritious meals for free during targeted occasions before expecting them to incorporate such dietary changes into their daily lives, which may subjectively be perceived as difficult	5	(75, 258)
38	Exaggerated health cautions concerning food ingredients or nutritional value should not overshadow the importance of a nutritionally adequate diet balancing macro- and micronutrients for overall health and well-being. While nutritional awareness is essential, excessive focus on specific nutrients or foods can lead to an unbalanced, unhealthy, and abbreviated diet, and overly restrictive dietary guidelines may harm mental health and cause disordered eating	5	(77, 93)
39	Maintaining the body's adequate balance in hydration is a fundamental prerequisite for healthy body functions, promoting cognitive function and mental clarity, preventing fatigue and headaches, regulating body temperature, and supporting healthy skin among other health benefits	1	(84, 90)
40	The factors that affect how people perceive and evaluate the potential risks and benefits of less-healthy food choices such as their knowledge, attitudes, and behaviors should be considered equally by both consumers and healthcare professionals	5	(71, 77)
41	Adopting long-term adherence to a healthy kind of diet can advantageously affect other lifestyle areas, emphasizing personal diet as one of the most critical lifestyle factors. Accordingly, it can serve as a determinant of continuation to other health-promoting behaviors, i.e., better sleep, increased PA levels, reduced stress levels/better resilience, and a lower likelihood of engaging in substance abuse, amongst other behaviors	1	(160, 177)
***Theme 3. Active Living (n** = **15)***			
42	Engaging in regular and consistent PA and health-oriented sports/exercise is crucial in enhancing and sustaining health throughout the lifespan	1	(146, 148)
43	PA and health-oriented sports/exercise habits not only enhance the physiological health of almost all organs but also can improve mental and psychological well-being and cognitive function, ultimately promoting an improved quality of life and greater success in occupational pursuits	1	(149, 150)
44	The components of a healthy lifestyle related to PA, sports, and exercise are determined by various factors such as exercise modality, intensity, frequency, duration, safety, and other parameters	1	(147, 259)
45	To promote health, it is recommended that children are active for at least 60 min per day, while adults should engage in 150–300 min of moderate-intensity or 75–150 min of vigorous-intensity aerobic activity per week, along with muscle-strengthening activities at least 2 days per week. Any full-body movement that incorporates all main muscle groups into tension is among the most favorable types of physical exercise for health promotion	1	(154, 260)
46	To establish a regular—ideally daily and outdoors/in nature—PA routine and maximize its health-related benefits, guidelines for weekly PA can be translated to a minimum of 20–30 min of moderate-to-high intensity workouts per day that includes both aerobic and intense anaerobic activities (including strength training). Concurrent training or alternating days is recommended to conserve good health status and to prevent developing health conditions	5	(177, 260)
47	Incorporating PA into daily routines as active mobility, such as taking the stairs instead of elevators and cycling or walking instead of driving, along with implementing other movement-based habits in weekly practices, such as household chores, gardening, or dancing, can be a practical means to achieve the recommended amount of PA	5	(183, 261)
48	Individuals who struggle to meet the minimum level of PA recommendations should consider starting with less intense activities and gradually increase the frequency, intensity, and duration of their training sessions over time	5	(153, 261)
49	Misconceptions about participating in sports and exercise can have adverse effects on specific health conditions, including asthma, hypertension, and joint problems. However, these beliefs may need to be scientifically supported and often arise from more understanding about choosing the appropriate type, intensity, duration, and frequency of PA, sports, and exercise, as well as the necessary precautions to take during exercise	5	(259, 260)
50	PA, sports, and exercise may potentially cause physical injuries, physiological distress, and even death if established procedures and standards are not followed	1	(183, 260)
51	To determine appropriate PA, sports, and exercise programs, especially for people with disabilities, regular monitoring and feedback analysis may be necessary due to a lack of research on the dose-response relationship between volume and intensity of activity in the target population	5	(259, 260)
52	Incorporating an active lifestyle can positively impact gene expression by decreasing the expression of unfavorable genes (especially those associated with obesity) and increasing the expression of beneficial genes, leading to significant benefits for future generations	1	(147, 150)
53	Developing public spaces that incorporate signage and features promoting a healthy and active lifestyle represents a potentially effective strategy to facilitate regular engagement in PA, sports, and exercise, while also encouraging healthier behavior among the public	5	(261, 262)
54	For patients at advanced stages of NCDs who face difficulties leaving their homes, a home-based PA regimen (such as seated yoga, geriatric, and/or strength training) can serve as a valuable tool to sustain a routine of healthy, active behaviors	5	(260, 261)
55	Due to the genetic diversity within human populations, individual variability, and environmental differences, it is essential to implement “holistic” and “personalized” strategies when developing physical exercise plans. These individually tailored strategies should consider each person's unique needs and characteristics to ensure optimal health outcomes	1	(165, 263)
56	Engaging in regular PA, sports, and exercise has the potential to extend multifaceted impacts on almost all aspects of lifestyle, pronouncing it as one of the most significant lifestyle factors. Accordingly, it can serve as a determinant of adherence to other health-promoting behaviors, resulting in improved sleep quality, adoption of healthier dietary patterns, cultivation of stronger social relationships, reduction in stress levels, and decreased likelihood of engaging in substance abuse	1	(160, 177)
***Theme 4. Other Health Behaviors and Related Dependencies (n** = **12)***			
57	Although a small amount of psychological and mental stress can be beneficial in terms of promoting mindfulness about health and enhancing overall productivity (referred to as eustress), excessive amounts of distress (generally perceived as unfavorable), especially when experienced chronically, can have adverse effects on both mental and physical health and overall well-being	1	(177, 264)
58	Effective strategies for managing psychological and mental distress and promoting overall well-being include regular engagement in PA, sport, and exercise, the practice of meditation, yoga, or other relaxation techniques, the maintenance of a healthy diet, minimizing the exposure to situations of negative stress and pressure, and seeking social support	1	(178, 264)
59	The negative effects of psychological and/or mental distress on cognitive function and decision-making abilities can compromise safety and increase the risk of errors and faults. Therefore, individuals in high-stress occupations need to prioritize stress management strategies and seek professional help if needed to maintain optimal performance and ensure safety	1	(51, 264)
60	Poor sleep patterns (including inadequate sleep quality, insufficient duration, and irregular timing) can have a significantly negative impact on lifespan and daily routines, including the maintenance of other healthy lifestyle areas such as diet, PA, stress management, relationships, and substance avoidance	1	(177, 265)
61	To promote healthy sleep habits, adults are recommended to get 7–9 h of undisturbed sleep per night, maintain a regular sleep schedule, create a relaxing sleep environment, and avoid stimulating activities before bedtime	1	(177, 265)
62	Sleep deprivation can significantly impair performance in occupations requiring high cognitive demand or memory allocation, such as pilots and flight attendants, surgeons, medical residents, nurses, emergency responders, shift workers, drivers, educators, and athletes. Therefore, prioritizing adequate sleep and rest for these cohorts is critical to maintain optimal health and productivity, potentially even more important than other health-related behaviors	1	(185, 265)
63	Strong and positive social relationships are associated with various health benefits, which are linked to social support, a sense of belonging, and positive emotions fostered by healthy relationships	1	(59, 184)
64	Positive social connections can have a favorable impact on the motivation to adhere to other health behaviors. In contrast, poor relationships and social isolation can be significant barriers and demotivating factors for maintaining a healthy lifestyle, especially in vulnerable age groups (e.g., children/adolescents, elderly) or among individuals who may already struggle with mental health conditions	1	(177, 184)
65	International recommendations suggest that healthy relationships with loved ones (e.g., partners, family members, friends, and colleagues) are crucial for promoting overall health and well-being. Furthermore, cultivating strong and supportive relationships with pets and other animals can also contribute to better physical and emotional health	1	(60, 177)
66	While the six pillars of lifestyle are important for promoting overall health and well-being, there are various other health behaviors that can help to improve physical, mental, and emotional health and contribute to a more fulfilling and satisfying life. These include activities such as managing screen time (especially in childhood and adolescence), practicing gratitude, engaging in artistic activities, spending time in nature, maintaining good hygiene, listening to music, receiving massage, and maintaining healthy sexual behavior	4	(60, 117)
67	If one specific lifestyle factor is violated by a poor or critical condition, it can have a remarkable detriment on one's overall health and well-being, even if the average state of one‘s lifestyle is appropriate and considered rather healthy at the same time. Therefore, it is crucial for individuals to pay at least basic attention to all health behaviors and habits in a more holistic and integrated manner to maintain their overall health and prevent unforeseen negative consequences	5	(59, 177)
68	The resilient nature of some lifestyle-related issues and problems, such as those associated with chronic stress, may not exhibit acute signs but can have negative impacts on a specific future state of health (e.g., in psychosomatic and/or psycho-neuro-immunological conditions). This should be considered when discussing the long-term health consequences of lifestyle choices	5	(16, 117)
***Theme 5. Health Education and Literacy (n** = **14)***			
69	The development of modules, courses, and trainings pertaining to preventive and promotive health strategies in medical and nursing curricula represents a vital endeavor toward equipping future doctors, therapists, and other healthcare professionals with comprehensive evidence-based knowledge and understanding the importance of preventive interventions. It also provides them with basic competence-orientated qualifications and skills ready to apply to practical settings, such as patient interview and consultation, which enables them to maintain as well as enhance individual and public health	5	(61, 187)
70	Providing robust and holistic health education from elementary to secondary school levels (competence-oriented qualifications and skills), which is in line with the state mandate in school curricula but not yet practically established, can be an effective approach with urgent priority to familiarize children and adolescents with healthy behavior (i.e., the willingness to act along with health action readiness). With this key prerequisite for future generations, comprising parents, teachers, school health professionals (i.e., physicians, nurses, etc.), decision-makers, and other members of society, the adherence to evidence-based health recommendations would naturally progress with positive cycles	5	(220, 222)
71	Integrating tertiary courses, modules, and trainings that address healthy behaviors and lifestyle choices into college and university curricula is a key approach to empower students (as future game-changers and/or leaders in various health-related fields, including policymaking) with evidence-based knowledge, including proficiency-oriented pedagogical and didactic skills, that results in a better understanding of the importance of lifestyle behavior and holistic health approaches as preventative measures	5	(187, 225)
72	The acquisition of thorough knowledge regarding healthy lifestyle behavior and integrating it into their personal lives is crucial for teachers, educators, trainers, lecturers, and academic staff. This enables them to effectively convey information to their pupils and students and empowers them to serve as authentic, genuine, and exemplary role models, making a significant contribution in shaping better future public health for future generations	5	(227, 228)
73	In line with the advancement of science, it is crucial to frequently update the knowledge base and training programs related to healthy lifestyle behavior for educators and healthcare providers in all educational settings, ranging from elementary to tertiary levels. This action is essential for promoting minimal well-being and preventative health measures and to ensure that educational content remains up-to-date	4	(109, 110)
74	Health education and literacy should not be confined to school classrooms alone, as practical environments like school gardens, kitchens, labs, general caterings inclusive buffet/canteen/cafeteria, can also provide valuable and additional learning opportunities	5	(220, 222)
75	Instilling skill-based lifestyle habits through playful and enjoyable methods is a highly effective strategy for motivating pupils and students to engage in similar health-promoting activities outside of their educational environments and settings	5	(187, 220)
76	A complementary competition-based approach (planned and implemented sensitive to age, school level, stage of development, and pedagogy/didactic tools), can effectively promote healthy lifestyle behavior among pupils and students. This approach adds an element of fun and motivation to the academic process by creating positive and purposeful skill-based occasions focused on PA, sports, and exercise, healthy eating, and other health behaviors, adding more potential to encourage adopting and maintaining healthy habits over a lifetime	5	(220, 222)
77	The establishment of stringent, health-oriented regulations for schools, colleges/universities, as well as public and private cafeterias, buffets, or dining halls with a mandate to provide diverse, healthy, and nutrient-dense WFPP meal options daily represents a key strategy toward promoting healthy dietary habits among pupils, students, teachers, lecturers/professors, and parents	5	(220, 229)
78	School and tertiary educational policies promoting PA, sports, and exercise should prioritize encouraging active mobility and transport to school for all to increase overall population activity levels. Specific attention should be directed to pupils with a risk of developing NCDs, such as overweight/obesity, insufficient PA, or a family history of such conditions	5	(266, 267)
79	Attentive and continuous monitoring of children and adolescents, particularly those with suboptimal health status, may motivate individuals in this age group to adopt a more conscientious, self-respondent, and independent approach toward their health-related behavior	5	(222, 250)
80	Institutionalized Sports Education (i.e., by sports clubs or commercial providers) represents a compelling approach to health promotion, which can be purposefully integrated alongside and independent from the compulsory school subject, Physical Education. This pedagogical approach encompasses the dissemination of sports culture, cooperative skills, technical skills-related knowledge, and joyful and competitive elements, collectively contributing to a well-rounded approach toward active living	5	(266, 267)
81	School principals and educators should consider the pupil's starting point and measure progress on an individualized basis. To this end, children and adolescents should be engaged in health and movement activities that are appropriate for their current level of ability and skills with a gradual increase in load as they advance toward higher levels of competencies and mastery	5	(220, 222)
82	School-based health and activity programs can further enhance their efficacy by affording pupils with a degree of responsibility, allowing them to assume the role of activity leaders and supervisors in the design, preparation, and execution of such programs	5	(220, 225)
***Theme 6. Research Priorities and Considerations in Health Behavior (n** = **19)***			
83	Interdisciplinary and multi-sectoral collaboration must be actively pursued in future research endeavors to develop effective, personalized, and comprehensive interventions, programs, and measures to promote more healthful and sustainable lifestyle behaviors and habits	5	(164, 268)
84	Future research should prioritize the health and well-being of vulnerable populations, including infants, children and adolescents, emerging adulthood, as well as individuals with a background of NCDs, by developing effective interventions, guidelines, and policies promoting their health behaviors. This prioritization should be done while considering the social determinants of health and ethical considerations for each specific group	5	(21, 269)
85	Further research should be conducted using more differentiated study designs to distinguish the specific associations of a particular health parameter calculated at different levels (e.g., poor sleep, acceptable sleep, healthy sleep) with the most critical health factors (e.g., diet, PA, stress, substance use, relationships). This will help to understand the magnitude and direction of the potential associations and inform the development of more targeted and effective interventions to promote overall well-being more comprehensively	5	(21, 269)
86	To accurately assess health behavior indicators, it is necessary to have more dependable assessments that span multiple levels, from very general health habits to corresponding biomarkers. This requires a comprehensive approach incorporating self-reported data, objective measures, and biometric assessments to holistically understand an individual's health behavior	5	(248, 269)
87	Future investigations should consider lower-prevalence lifestyle-related populations (such as vegans and vegetarians), as they have been shown to have various direct and indirect impacts on other lifestyle behaviors. Proper and up-to-date classification of these populations based on international references can lead to a better understanding of the health landscape and result in more effective healthy lifestyle approaches	5	(74, 248)
88	To uncover health consequences before severe health conditions manifest, it is essential to monitor trends in each health behavior from mid- to long-term while considering statistically confounding variables and linking them with other subjective health behaviors and objective determinants at various stages of analysis. Therefore, it is crucial to develop multi-component and multi-level study designs that enable the measurement of confounding effects rather than solely adjusting or controlling them in data analysis	5	(164, 270)
89	Further research is necessary to investigate the potential indirect changes in healthy lifestyle behaviors arising concurrently or subsequently to other health behavior interventions. Analyzing the potential indirect effects of health behavior interventions can also help identify unintended consequences that may hinder the success of health promotion efforts, such as the displacement of one healthy behavior by another or the adoption of unhealthy compensatory behaviors	5	(60, 175)
90	An up-to-date scientific consensus considering the latest research with short-term search-and-test intervals is necessary to establish standardized cut-off points that can be utilized to differentiate the distinct levels of each lifestyle behavior, ranging from healthy to unhealthy leveling	5	(21, 269)
91	Considering the strong interconnection between diet and PA, as they both constitute components of the energy balance equation, it is essential for research efforts to consistently investigate these two domains in a cohesive and integrated manner, considering their inextricably interwoven nature	5	(168, 261)
92	Considering the importance of the family in developing and solidifying children's health behaviors, there is a necessity to establish well-controlled research studies that can foster empirical understanding of parent-mediated variables in populations of children and adolescents, as well as peer groups in emerging adulthood	5	(175, 269)
93	As technology dominates educational practices, there is an urgent need to frequently update and enhance relevant research data and guidelines, particularly with regards to technology-related health behaviors, such as the educational and non-educational aspects of screen time use among children and adolescents	5	(28, 247)
94	Additional research is necessary to investigate the monetary burden and financial consequences of unhealthy lifestyles and the economic advantages of health promotion programs. This requires differentiated assessments for each of the six lifestyle areas, as well as for the holistic lifestyle medicine approach, and can aid in the development of an economic perspective on lifestyle behaviors that trace from better personal health to improved future public health	5	(48, 254)
95	Research on designing, updating, and validating tools and manuals to measure health behaviors is crucial to ensure reliable and comparable data collection, identify patterns and trends in health behaviors, and evaluate the effectiveness of interventions and policies aimed at promoting healthy lifestyles across diverse populations and contexts	5	(250, 269)
96	Future research should focus on understanding the practical settings in which people engage in daily activities such as work, transportation, and leisure time to identify opportunities for promoting health behaviors in an effective and efficient manner. Therefore, it is important to explore how these settings may impact individual's health behavior and to develop tailored interventions that consider personal needs and nature to promote their health without placing undue burden on time and energy resources	5	(254, 269)
97	To better understand the link between exposure and outcome variables over time, future research should prioritize large observational cohort studies, alongside RCTs. These studies gather longitudinal data and measure multiple factors related to health behaviors, revealing patterns and trends in health outcomes and establishing causal links between risk factors and health outcomes. Larger sample sizes also increase statistical power, improving the accuracy and reliability of the findings	5	(21, 269)
98	Innovative and fit-for-the-future research methods (such as advanced and human primary or stem cell-based applications, organoids, organ-on-chip-models, artificial intelligence, and 3D printing of standardized, complex human tissues) following robust study designs and targeting specific human populations and the etiology of each chronic disorder should supersede animal studies that potentially yield spurious results	5	(30, 247)
99	As technology rapidly evolves, future scientists and policymakers should explore the potential benefits and limitations of technology in personalized medicine to enhance personalized healthcare delivery, including prioritizing the development and integration of cutting-edge technologies such as artificial intelligence and machine learning, as well as e-health tools, devices, and services	5	(30, 32)
100	To effectively integrate multiple types of data to understand individual and population health, it is recommended that world-leading policy and health organizations prioritize the development of interdisciplinary collaborations and data-sharing frameworks, while also investing in advanced analytics and innovative technologies	5	(237, 267)
101	Collaboration of policy- and decision-makers with research teams can promote evidence-based initiatives, allowing for the dissemination of up-to-date health information that is appropriately tailored and prepared for specific target groups within societies and national populations. This collaboration is also essential for the acceptance and implementation of new approach methods without the use of animal testing or experimentation in chemical and drug regulation/legislation	5	(271, 272)

PA, physical activity; NCDs, non-communicable diseases; WFPP, whole food plant-predominant diet; RCTs, randomized controlled trials; Suppl. Sources, Supplementary Sources.

## 4 Overview and discussion

### 4.1 Summary

The three international congresses of research and knowledge exchanges between 2020 and 2022 aimed to contribute evidence-based consensus statements to address today's global health dilemma (rating quality of evidence see Table 1). These congresses were held to improve the individual's optimal state of health, which would cumulatively reduce the social, ecological, and monetary burden of chronic diseases in the mid- and long-term, ultimately resulting in better public health on a global scale. Considering the limitations and neglected gaps in current health-related approaches, this panel has proposed practical strategies that prioritize a holistic and integrated health perspective, specifically focusing on key elements of sustainable health behavior outlined in this report. Accordingly, research priorities and policies for future scientific and practical efforts have also been suggested.

The evidence-based statements developed by this consortium encompass various topics concerning sustainable health behavior and the improvement of global health, including general considerations on and approaches toward a good state of health (theme 1), the minimum (primary, key) behaviors associated with good health: “Healthy Eating & Active Living” (themes 2 and 3), other important, non-energy-balance-mediated lifestyle behaviors (theme 4), health education and literacy as critical aspects associated with good health and wellbeing (theme 5), and research priorities (theme 6). Regarding basic approaches, the statements (*n* = 22) generally emphasize the value of addressing health behavior as a complex and dynamic phenomenon shaped by individual, social, and environmental factors. This requires a focus from individual-level interventions, such as goal setting and self-monitoring, to broader population-level approaches that aim to change health-related social norms and policies concerning care. Regarding healthy eating, the statements (*n* = 19) highlight the importance of an adequate healthy diet considered medicine for various lifestyle disorders/conditions. Strategies for promoting healthy eating emphasize whole-food, plant-predominant diets and include nutrition education and policy initiatives such as subsidies for healthy foods and restrictions on marketing unhealthy products. For active living, the statements (*n* = 15) emphasize the importance of regular PA for improving and maintaining good health and reducing the likelihood of the associated risk factors. Strategies for promoting PA include environmental and policy changes, such as improving access to safe and convenient places for PA engagement. Other health behaviors addressed in the statements (*n* = 12) include sleep, stress management, relationships, and substance abuse. These areas highlight the need for a holistic and contemporary approach to health that addresses a cognizant range of lifestyle factors that contribute to overall health and wellbeing. In health education and literacy, the statements (*n* = 14) emphasize the need for effective communication and health literacy skills to promote behavior change and informed decision-making. This area includes the importance of tailoring evidence-based health information and messages to individual needs and preferences, ensuring its accessibility and distribution at all educational levels. Finally, the statements highlight the importance of research in advancing our understanding of health behavior and informing effective interventions (*n* = 19). This includes the need for interdisciplinary collaboration, rigorous methodology, and consideration of various critical factors in research designs and implementation. Overall, the 101 statements provide an in-depth overview of the essential de-medicalized factors involved in promoting and maintaining good health through behavior, with a particular focus on the dual approach of sustainable and lifelong health and wellbeing and the associated educational and communal considerations on the simple formula of “Healthy Eating & Active Living.” By addressing the complex and multi-faceted nature of health behavior and highlighting effective strategies for promoting behavior change, these statements offer valuable insights and guidance for researchers, practitioners, decision-makers, and policymakers in the field of public health.

### 4.2 From individual to public health

**Figure 4:**
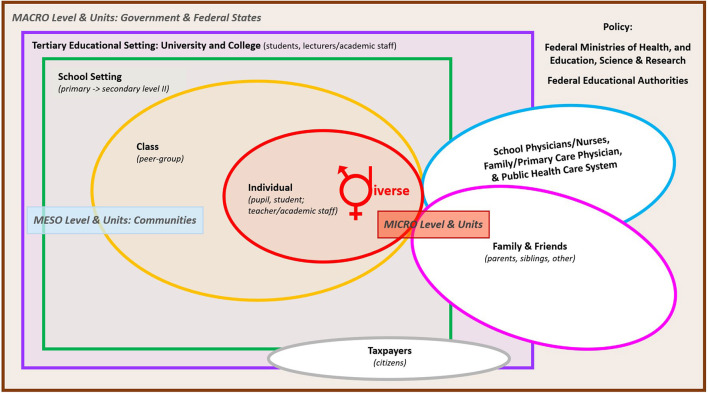
Target groups considering personal and public health to be addressed, from the micro-level of individuals and families to the meso-level of communities (e.g., schools, universities, regions), to the macro-level determined by state and federal policies; in accordance with “The Main Health Determinants Model” by Dahlgren and Whitehead (252, 268). © Katharina C. Wirnitzer.

The dual approach of “Healthy Eating (at best, WFPP, and preferably vegetarian/vegan) and Active Living (at best daily and outdoors/in nature)” is a scientifically informed strategy that is well-known as promising due to its unique health benefits (13–15, 155–163, 165–172). The approach emphasizes the codependent and continuous application of healthy dietary patterns and regular engagement in PA, sports, and exercise, which serves to address the dynamic interplay between energy intake and expenditure (163, 238). By avoiding the development of NCDs and the risk factors, the dual approach is increasingly recognized as a cornerstone of preventive medicine, public health, and care practice. It highlights the significance of a holistic perspective that integrates various dimensions and interactions to generate network models of health and disease functioning (43, 45, 167, 273). Therefore, “Healthy Eating & Active Living” displays the minimum recommendation to unlock and begin tapping into the full potential of the power of lifestyle organization and change to improve one's health at the individual level from childhood into adulthood and old age (50). This, in turn, can contribute to prioritizing the person (and/or patient) centered approach over disease (and/or medication-) centered and thus shaping better public health of nations. Figure 4 displays the most relevant target groups in accordance with and derived from models of the determinants of health (29, 179, 180, 252, 268). With the potential to shift from a patient- and disease-centered approach to a preventive person-centered health approach, the model spans all levels and units of action to achieve better public health of nations that emerge from improved individual health.

**Figure 5:**
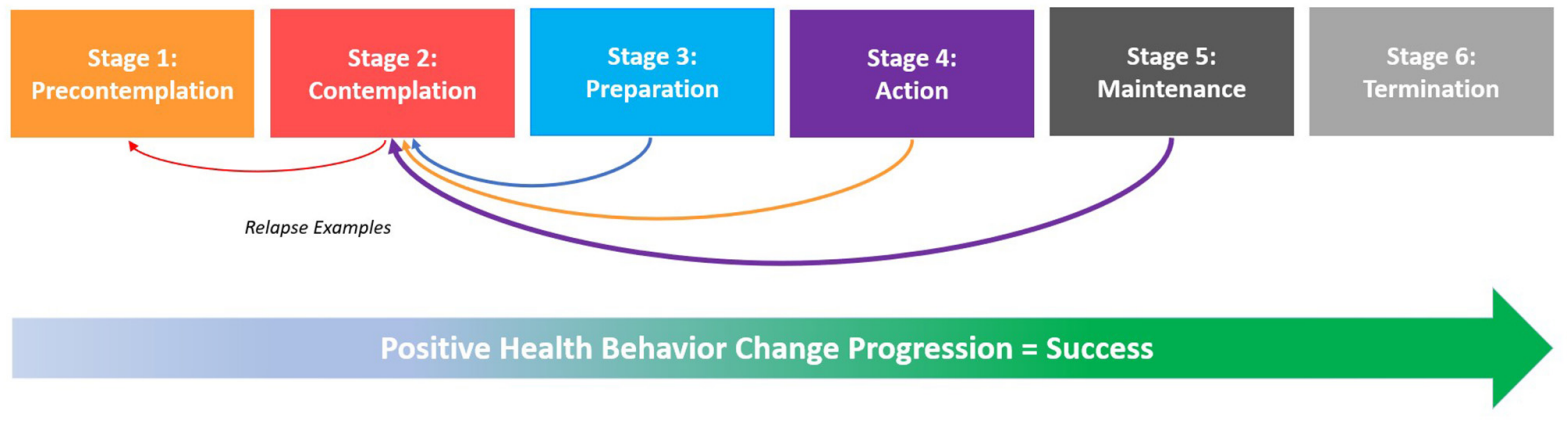
Stages of Behavior Change Theory Model within the challenge of the behavior change process of adulthood. Graphic illustration created on Raihan and Cogburn (181) and Prochaska and Velicer (182) © Derrick R. Tanous/Katharina C. Wirnitzer.

Consistent with the saying “you can't teach an old dog new tricks,” childhood, adolescence, and emerging adulthood are extremely sensitive life stages for ingraining healthy behavior. However, the challenge of the behavior change process remains significant for adults to cope with (Figure 5), and considering that relapses may occur (and at various stages), the successful adoption of new healthy behavior and the complete termination of previous unhealthy behavior is rare (Stage 6). Therefore, defining behavior change success is suggested to be any forward progression in the model rather than focusing solely on reaching the final stage of termination. Indeed, when the power of personal behavior at its lowest range estimate (the cause of 40% of all deaths) exceeds all other determinants of health (29, 30), including the health care system (10%), environment (5%), social circumstances (15%), and genetics (30%), the pursuit of addressing this challenge becomes even more crucial.

### 4.3 Current and future health approaches

The introduction of novel medications and drugs stimulates economic growth, creates employment opportunities, and contributes to the overall success of the capitalist system (274, 275). As a matter of fact, it is valuable for companies, especially in terms of profitability, to prioritize the continuous treatment of individuals with long-term health issues using modern medical products, rather than addressing preventive and sustainable health behavior across different societal levels—a challenge often encountered in the field of medicine (275–278). This consequence is especially apparent when comparing the impact of different health determinant domains on money spent in billions annually, e.g., behavior has a 38% annual impact (equivalent to USD 260 billion), while medical care has an 11% annual impact (or total USD 3,337 billion) (29). The mainstream media may play a key role in investigating and promoting contemporary medical activities while neglecting the primary mechanism of NCDs (274). In today's case, industrialization is successful with its crucial strategy of hidden and unregulated attempts to change public perceptions about health and illness (276–278). In Europe and the US, prescribed medications rank as the third leading cause of death right after heart disease and cancer, with approximately half of these deaths due to taking the medication correctly as prescribed, while the other half of these deaths are due to overdoses or drug abuse (274, 279). Interestingly, around 70%−80% of psychotropic (including antidepressant) medications are prescribed in primary care practices (280). The adverse combination of medical universities aligning closely with their university-industry partnerships, and the emphasis on patents rather than public-interest science, is quite evident (274). Often, the industry's conflict of interest is apparent rather than subtle since disease prevention, non-drug-dependent methods lack commercial interest, as healthy people have little-to-no dependency and are not lucrative clients for the medication industry (279). Healthcare and medicine must evolve into a proactive public health system that is predictive, preventive, personalized, and participatory (P4 model) (263). Therefore, it is crucial to step back from a fragmented and merely reactive health approach, which involves minimal interactions among specialists, often resulting in non-concerted prescriptions and scattered follow-ups. This approach also limits the information available to primary/family care physicians, therapists, and individuals/patients, ultimately leading to a suboptimal cost-effectiveness ratio. Along with the Hodges' model (a comprehensive framework offering a practical approach for learners and professionals considering health and aligned with the SDGs) that navigates healthcare complexities, transcends boundaries, encompasses various literacies, and fosters interdisciplinary connections (273), the integrated “P4 Health Spectrum” model promises to reduce the significant burden of NCDs and increase healthy aging and the health span (263).

Currently, the public health community partially relies on commercial determinants of health, specifically factors influenced by profit motives and monetary gain (281). In fact, many countries with tax-financed public health systems are increasingly facing social and health care inequalities due to profit and privatization-oriented health care reforms (282). In the ongoing conflict concerning the detrimental impact of commercial interests on health, particularly advertising strategies used by tobacco, alcohol, processed food, and sugary beverage entities, it's important to note that these influences drive (and often control) not only what to eat and drink but also individual access to health services (29, 180). It is well-documented that the process of ultra-processing food is predominantly associated with a loss of its nutritional value (283, 284). Currently, in the USA, Canada, and the UK, half or more of dietary energy comes from ultra-processed food products, and the production and consumption of these products are rapidly increasing worldwide (276–278). When introduced into young people's lives, for example, sugar-sweetened beverages contribute significantly to the development of overweight/obesity in children and adolescents (243, 285, 286). Obesity can cause severe diseases in childhood and adolescence and is a risk factor for obesity, cardiovascular morbidity and mortality, diabetes mellitus, and other chronic diseases in adulthood (287). Evidence indicates substantial health benefits associated with improving nutrition during the early stages of life, including the promotion of breastfeeding and the improvement of the quality of early childhood feeding (288). Eating habits, mainly shaped by the domestic environment, often persist throughout life (257). Adopting a healthy eating approach can positively affect other aspects of lifestyle, resulting in an overall healthier lifestyle, and can practically serve as a key factor in the transition to and adoption of other health-promoting behaviors, such as better sleep, increased PA/exercise levels, reduced stress levels/better resilience, and a lower likelihood of engaging in substance abuse (72, 76–78).

**Figure 6:**
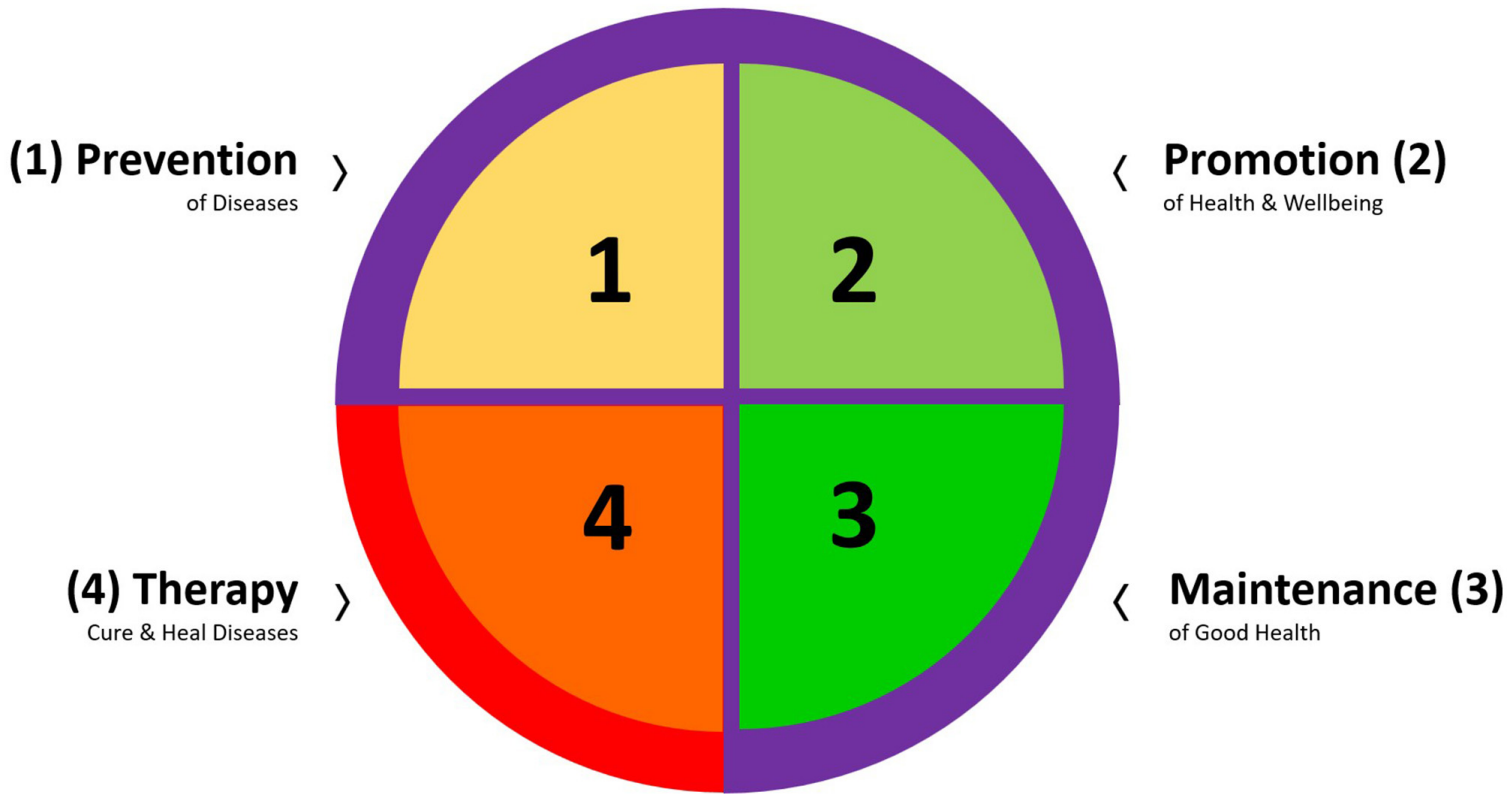
Four areas of action comprising health-oriented action, competence, and readiness in order to achieve sustainable and lifelong health and wellbeing. © Katharina C. Wirnitzer.

By embracing such an effective de-medicalized approach to health (i.e., “Healthy Eating & Active Living”), preventive and therapeutic models can surpass empirical reductionism (253, 289). To meet this, it seems crucial for any therapeutic approach to consider and effectively apply the three-to-one ratio (Figure 6): three competence-orientated areas of health-related actions, including—first and foremost—the prevention of diseases, maintenance of good health status, and promotion of health, followed by one, as a last resort, medicalized therapeutic strategies (as a fourth area of competence-orientated actions) to target specific health conditions, including curing and healing diseases. Consistent with this “prevention first” appeal (182, 184), EU policymakers have already identified and emphasized (i) the urgent need for greater and more pressing efforts toward a shift to the prevention of ill-health and disease with health promotion as a key pillar in ensuring future public health (114, 118), and (ii) the cross-European need to focus on changes in future medical and health pedagogy to address the current public health gaps (262, 290).

In line with the statement of Dr. Anthony Fauci, the chief medical advisor of the US, “the first thing is to stick with the science” (interviewed by the NEJM); therefore, it is imperative to follow the conclusive evidence on healthy lifestyle behavior when considering disease prevention, health maintenance, and the promotion of good health altogether (291). Sticking to the science, however, is long overdue in the promotion of better public health of nations worldwide, except for the science of a specialized therapy, especially when dealing with preventable diseases, which, in such cases, intervention is often too little, too late. A current Lancet comment states that it is not enough to talk about change, and thus tertiary entities need to act in helping society to grow and tap its potential for change (292). As crucial platform not only to empower future health-professionals but individuals for a healthier life, too, colleges and universities play a pivotal role in establishing the provision and transition to evidence-based information about the health-related power of lifestyle medicine and lifestyle behaviors as foundation for informed lifestyle choices. In this regard, universities must advocate for the plant-predominant dietary transition as one key pillar of empowering people for a healthy life, starting with students and academic staff (292, 293). Since the COVID-19 pandemic, there is an ongoing debate regarding the question of how firmly scientific findings and recommendations for primary care practice are rooted in high-quality, research-based evidence (294–296). It has been reported that only 18% of the recommendations for primary care practice are based on patient-oriented evidence from consistent, high-quality studies (294). Consistently, findings from a study assessing changes in the quality of evidence in updates of Cochrane reviews indicate that only a minority of outcomes for healthcare interventions are backed by high-quality evidence (9.9%), and the quality of the evidence does not consistently show improvement or deterioration in updated reviews (251). Another study indicates that over 90% of healthcare interventions examined in recent Cochrane Reviews lack robust support from high-quality evidence, and there is insufficient reporting of associated harms (297). Moreover, in various scientific domains, research findings are frequently presumed to reflect the prevailing bias (298), and a significant number of published results may be false or exaggerated (277, 278, 299–301), with an estimated 85% of research resources being wasted (302). However, it is inconceivable to envision modern healthcare without considering evidence-based medicine (269), and readers of journals should exercise caution when interpreting research findings (303). The adoption of large-scale collaborative research, standardization of definitions and analyses, pilot examination of interventions, and improvement in study design standards, peer review, reporting, and dissemination of research have been identified as crucial actions to enhance the validity of scientific works (302). The recent planning of the Evidence Synthesis Infrastructure Collaborative is a great example of aiming to combine evidence synthesis from all fields of research and all countries globally (304). The planning phase was run by Cochrane, Campbell and the Joanna Briggs Institute (Australia) and the execution will start in the fall of 2025 by Wellcome UK, investing 45 million GBP in establishing this global infrastructure. The aim is to work toward better informed decision and policy-making, with the Global South in the lead and sustainability as the major focus point.

### 4.4 Scientific and educational animal use

Since “man is not a mouse” (305, 306), an advanced, fit-for-future, holistic, and de-medicalized health and care approach requires methods free of animal testing, as animal experiments no longer meet today's bio-technological standards regarding the advancement of medical and human sciences. In line with the well-documented incompatibility between animal experiments and human trials (209, 307, 308), current evidence from systematic reviews identified significant limitations in preclinical animal research, encompassing both the quality of publications and translational value (209, 210). Translational percentages (concordance rates) of results exhibit a wide range from 0–100% with no predictable factors for translational success or failure in humans (309), and even multiple experiments fail to significantly contribute to improving the reproducibility of animal testing, accounting for 41%−72% of the observed total variance (310, 311). These results show how misleading animal experimentation can be in terms of human health and emphasize the necessity to study the specific human disease in detail first and, on this basis, select the cost-appropriate, technologically advanced human-relevant model or method (e.g., use of isolated cells and tissues, organoids, robotics, computer bio-modeling) and system of testing before starting the research (312).

Recent decades have shown a tremendous development in science and technology, which have also made it possible to move to more human-centered science. Despite the challenges of the COVID-19 pandemic, it was possible to develop reliable vaccines for the market within 1 year, with fewer animal studies, increased use of alternatives, and expedited movement to clinical trials (272, 313). It should also be considered that legislative requirements for animal studies lack a solid, evidence-based foundation (314). In addition, there is a special need to entrench advanced alternatives across tertiary curricula and training for medical students, as well as students of relevant life and health sciences. Results from a systematic review on the educational efficacy of humane teaching methods indicate that learning outcomes are superior (30%), equivalent (60%), or inferior (10%) to those produced by traditional animal use (315). The review concludes that widespread implementation of humane teaching methods would preserve learning outcomes and may provide additional benefits for students and educators, tertiary institutions, and entities, with a particular focus on the wellbeing of animals (315). Animal-free methods in educational training and research, as part of de-medicalized future approaches to health, are on the pulse of time, indeed. With this, humane teaching methods have already been implemented within life and health sciences education and in many countries. Nowadays, ethically motivated students are able to pursue their studies in tertiary education and training without harming animals (316–318).

Over the past two decades, funding agencies have launched opportunities and specific calls for innovative projects across various disciplines to develop alternative methods replacing animal experiments (319–322). These initiatives and programs, often backed not only by national funding agencies and/or corresponding federal ministries but also increasingly on a European and world-wide level, aim to support the development of effective, efficient, human-relevant and human-centric technologies and tools in sustainable biomedical research (319–321). The recent launch of the new Ombion Center for animal-free biomedical innovations is a demonstration of major investments by the Dutch government to build an infrastructure offering research, education, training, advice and support to enhance the acceptance and use of animal-free biomedical innovations (323).

In this regard, innovative and award-winning databases provide transparent information, helping to avoid or at least reduce the use of animals in experiments characterized by repeated trials and multiple testing (324, 325). A recent survey commissioned by the Eurogroup for Animals, polled approximately 11,000 EU citizens from eight member states on the abolition of animal testing in research, testing, and education, with 77% of EU citizens in favor of phasing out animal testing (326). This outcome is in line with a registered 2023 European Citizens' Initiative advocating for a Europe without animal testing, supported by more than 1.2 million valid signatures from citizens of all 27 EU member states (327, 328), providing a strong indication of the shift away from animal testing.

### 4.5 The COVID-19 pandemic

Opportunities to follow healthy lifestyle behavior may be influenced by limiting factors and acute events that are beyond an individual's intention and control. During the past 5 years, COVID-19, as a magnifying glass of public health problems, posed an unprecedented worldwide challenge and evolved into a sudden public health emergency overnight, which is unparalleled in contemporary history (329, 330). To control the spread of the pandemic, social contact restrictions (e.g., encouraging people to stay at home, avoid non-essential travel, and maintain social distance) and lockdowns with (self-)quarantine (e.g., closure of public gardens, educational and recreational services and utilities) have been frequently implemented (331, 332). Regardless of the health-related problems associated with direct infection with SARS-CoV-2, data show that the pandemic has had unfavorable impacts on lifestyle behaviors (including dietary and activity patterns) and health status (including mental health) of various populations worldwide (333–335), which have gradually become more common than the outbreak itself. Additionally, education and community life have also been markedly affected by the COVID-19 pandemic and associated restrictions (336, 337), which may potentially result in further detrimental health effects from a long-term viewpoint (although this is currently hidden). Evidence indicates that healthy lifestyle behaviors, particularly regular engagement in PA, sports, and exercise alongside a healthy (particularly, WFPP, preferably vegetarian/vegan) diet can strengthen the immune system and reduce the likelihood of COVID-19 infection (338–344). Additionally, an active lifestyle is considered an effective strategy to reduce the psychological distress associated with the pandemic and lockdowns (345–347). During such an unprecedented pandemic, where medical and/or pharmacological treatments are not yet available to effectively manage its spread, a collaborative effort between health organizations and governmental decision-makers is necessary to promote evidence-based, de-medicalized approaches, such as “Healthy Eating & Active Living,” to prioritize providing practical incentives for people to consume healthier food options and engage in desirable PA, sports, and exercise opportunities (348–351).

Currently, although it appears that the world is in the final stages of the COVID-19 pandemic, there exists no guarantee that hidden health-related concerns (especially those associated with psychosocial health) will not potentially appear or develop in the form of post-pandemic consequences, or that the world will not experience a similar health tragedy in the future. While current global policies face challenges in effectively proposing and addressing Earth's overpopulation as a general approach to mitigate most global pandemics, there appears to be a further emphasis on the fundamental adoption of healthy lifestyle behaviors as a key factor in modern and sustainable health and care practices for better public health outcomes to address existing public health concerns. Nevertheless, the COVID-19 pandemic revealed that the health of the individual is not equal to the health of the community. Therefore, at the very least, benefits from behavioral change occur across societal levels. From the perspective of the unenthusiastic population health developments worldwide, as well as the COVID-19 crisis recently passed, the public health community urgently needs advice-focused, hands-on actions along with the drivers of those generating increasing social, health, and care inequalities. It requires the willingness to act from professionals of all health-related areas and spheres of influence to tackle inequalities and strive for fairer treatment in the post-pandemic world (180, 191).

Due to the positive health effects in the promotion and maintenance of health over the life course, the following observations shall be considered for the protection against moderate-to-severe COVID-19 infections during situations of severe health conditions (although they were not entirely present in the public mass media as general recommendation to aid in one's state of health or even to support vaccine efficacy): the risk for severe COVID-19 outcomes and the associated death was lower (i) in active people meeting the PA, sports, and exercise guidelines during the COVID-19 situation compared to inactive people (338, 352–362), (ii) in people following dietary patterns characterized by high-quality choices, especially through healthy plant foods as “best buys” (339) compared to those did not prioritize their diet quality, and (iii) in people following vegetarian and vegan diets, which were associated with lower risk and severity of COVID-19 (reduction of up to 73%) compared to those following omnivorous diets (339, 340, 359–367). To reduce the likelihood of future epidemics, positive lifestyle changes (i.e., toward healthy eating habits) must be reflected since evidence indicates that slaughterhouse cooling chambers provide an optimum environment to preserve highly infectious viruses, like SARS-CoV-2, which further raises the need to talk about meat (368, 369). When a high-quality diet (indicated by a high percentage of plant foods or entirely WFPP diets at best) is supported by regular PA, sports, and exercise (daily outdoors/in nature at best), the unique synergistic effects improve the cardiovascular, metabolic, and immune systems, which ultimately benefits immune-surveillance and has the potential to counter similar virus infections and symptoms at three preventative levels: (1) strengthening immune system in combating infections; (2) increasing vaccine-efficacy via responses from other types of infections (e.g., influenza); (3) improving physical fitness and quality of life. Thus, PA, sports, and exercise combined with a healthy diet serves as an effective and promising dual approach to protect the human body from similar viral infections and symptoms (338–365). Since current policies have insufficiently addressed people's willingness to change, it is time for public-health communities, decision-makers, and health experts to encourage people and patients on “Healthy Eating & Active Living” through professional guidance (370, 371). From this perspective, it is any physician's responsibility to prioritize individual, public, and global health issues (191, 246), as basal and permanent moral drivers in medical and health-related professions, not only limited to times of severe crisis.

### 4.6 Closing remarks

According to the ACLM (60), there are several key behaviors, including core competencies with the minimum of the dual approach to sustainable health:

- Eating healthy food before taking medication (i.e., Food over Medicine), and continuously linked to
- Moderate-to-high levels of day-to-day PA, sports, and exercise (i.e., Exercise over Medicine).

There is no evidence-supported doubt that diet is a major determinant of health and wellbeing (for good) or disease (for worse) (27, 372, 373). Although analyses and recommendations of renowned studies like The Multiple Risk Factor Intervention Trial (270), the North Karelia Project (374) and The China Study (375, 376) vary, the general conclusions of these and many other conclusive publications usually state that healthy dietary patterns are mainly plant-predominant. Regarding Food over Medicine, the term WFPP diet has been coined by pioneers in the field of healthy eating (95, 377–379), as plant foods are believed to be even more effective than medicine (103). Exercise over Medicine, however, shows that healthy diet also has limitations, as other aspects of life and behavior fulfill the holistic concept (172, 183, 380, 381). Physical education as a compulsory school subject, therefore, plays a greater role in school health promotion than any other school subject such as science, history, or math. From a developmental perspective, PA requires children to have competence in motor skills to be able to move effectively and safely (382), and therefore, it requires fitness development (266, 267). Indeed, the integration of school subjects as a cohesive body of knowledge emphasizes the significance of their interactions, reflecting the potential to promote healthy behavior (383, 384).

Data from epidemiological evidence, such as the EPIC-Oxford Study or the Adventist cohorts, show quite broadly how a healthy lifestyle, including PA and exercise, along with plant-predominant (including vegan) diets, can contribute to long-term sustainable health (100, 385, 386). That is to say that these populations have provided a basic example of a healthy aging lifestyle, which has been shown to expand the lifespan from 10–17 years (91, 387, 388). Nearly 700 million years lived with disability were caused by NCDs in 2013, which constitute 9 out of the 10 leading causes of years lived with disability (389). NCD-caused years lived with disability contribute to most of the disease burden in many countries (389–391) and insurance groups are advocating for healthier behaviors, especially vegan nutrition, due to the exceptional characteristics (392–398). Many countries are now spending 30%−86% of their health care budgets on the medical management and symptomatic relief of NCDs and other chronic conditions (18, 20, 254, 399). Times of increased stress levels come and go but remain prevalent for specific populations, as not all diseases are preventable (e.g., due to genetics or that many people currently live with NCDs/chronic conditions), and therefore stress management and relief practices (e.g., Red Noses Clowndoctors, Sensory Room Therapy, yoga) hold precious value (400–402). These holistic practices are closely tied to our interpersonal relationships, which profoundly contributes to mental health (264). Happiness, after all, is the highest goal for many people in life (403), with much to learn from the circumstances in Bhutan, the one and only country with a federal ministry on happiness (404). Without health, considered the greatest wealth, the potential for happiness experienced over a lifetime is restricted (405). Therefore, it is especially important to consider the scale of misery for individuals as well as nations (403). In the best-case scenario for individuals, starting from natural health at birth to the full extent of leading a complete, healthy lifespan, the end of the aging process almost guarantees discomfort and/or some degree of suffering (388, 390, 406), while the choices to behave accordingly to avoid additional problems remain. The development and implementation of comprehensive public metrics to evaluate healthy lifestyle behaviors, along with offering financial incentives, compensation, or bonuses from statutory health insurance funds to families, educational entities at all levels, employees, and businesses that adopt health-promoting behaviors, could be effective toward fostering a culture of health and wellbeing within society and across nations (8, 10, 146, 230, 231).

Considering the “prevention first” appeal (that has been acknowledged across many global health policies but has deficient implementation) and given the importance of informed lifestyle choices as a practical step (114, 172), health experts, policymakers, journalists, and patients should be conscious of corporate-sponsored material regarding the nature or prevalence of the disease. Instead, they should rely on publicly funded sources that provide information on the de-medicalized treatment of common health problems as a primary preventive course of action. Likewise, rather than relying solely on prescribed medication, it is essential to consider the power of lifestyle medicine and health behavior, prioritizing a person-centered approach to health over a disease-centered one (15, 167). This involves making healthy lifestyle choices as the first line of intervention, placing them above the immediate prescription of medications to address unhealthy behaviors. Collectively, it is also essential for politicians, physicians, and the public to address the disparities and conflicts between public health and private health, including issues like the right to health, a patient's bill of rights, and patient autonomy. Rather than debating slogans, it is crucial to identify tailored solutions to the pressing health issues and challenges (407).

In the context of holistic support of personal health (particularly the de-medicalized evidence-based concept of lifestyle medicine) and based on consensus that food and physical exercise are considered medicines, the basic and simultaneously sound dual approach that forms the foundation to sustainable and lifelong health and wellbeing is “Healthy Eating & Active Living.” Based on the interwoven, related, and integrated nature from both the key pillars of health, (1) healthy–at best WFPP, preferably vegetarian/vegan–diets permanently linked to (2) regular–at best daily and outdoors/in nature–PA, sports, and exercise (13–15, 155–159, 161, 162, 165–172) is the minimum recommendation to start tapping the full potential of the power of lifestyle to improve one's health on an individual level. “Healthy Eating & Active Living” must come first in school education and be seamlessly linked with college and university education to promote the health and wellbeing of nations; person- (or patient-) centered approaches trumps disease- (medication-) centered approaches.

## 5 Conclusions

Despite increased healthcare budgets and improved quality of scientific data resulting in the development of evidence-based guidelines and recommendations aimed at promoting better individual health outcomes, the prevalence of unhealthy lifestyles, chronic conditions, and environmental challenges continues to accelerate. This is particularly evident in industrial nations and Western societies, but it is also becoming more prevalent and alarming in developing countries. These trends suggest that global health efforts may not be as effective as intended in promoting better individual health outcomes to improve public health. The overemphasis on medicalization and the limitations of current treatment methods in modern, high-tech medicine in tackling NCDs raise serious concerns and pose major challenges in achieving sustainable healthcare in an increasingly digitalized world. Additionally, various perspectives on scientific conclusions in the commercialized areas and fields of the global health sector can further complicate the understanding and dissemination of appropriate health information. These perspectives may be influenced by interests that do not always prioritize people's health and can damage the credibility of experts, reduce patient confidence, and create significant obstacles to public health promotion efforts. As a result, it is important to recognize and address these challenges to ensure that accurate and reliable health information is accessible to all.

The present consensus paper summarizes the findings and conclusions of three multidisciplinary meetings held in Innsbruck, Austria from 2020–2022, which collectively hosted 284 internationally recognized experts from 76 universities, stakeholders, and organizations from five continents. The aim of these research and knowledge exchange events was to address today's unenthusiastic and challenging global health dilemma by sharing practical and purposeful strategies to develop healthy lifestyle behaviors and habits, with a special focus on the evidence-base of “Healthy Eating & Active Living,” at best WFPP, preferably vegetarian/vegan diets permanently linked to PA, sports, and exercise at best daily and outdoors/in nature, as the minimum recommendation for initiating advantageous personal lifestyle changes for health. The goal was to motivate and empower individuals, general populations, as well as health, education, and policymaking experts alike to tap a huge intrapersonal potential often neglected or ignored. The experts identified gaps in health-related approaches, addressed how to tackle and bridge these, and debated research priorities and necessary policies for future efforts. The consortium of authors made and approved a significant number of consensus statements (*N* = 101) spanning across six specific themes based on sound and robust scientific evidence.

In conclusion, to address the current global health dilemma of today's high-tech medicine, multiple strategies are necessary, including establishing innovative curricula at all educational levels for children/adolescents, future teachers, pedagogics, and healthcare professionals. This should be accompanied by increased stakeholder participation from policymakers, health authorities, and public bodies, tackling organizational challenges related to multidisciplinary collaborations, addressing infrastructure requirements, revising disease classification systems, updating testing models, establishing regulatory frameworks and reimbursement models, and addressing potential ethical, legal, and social concerns. Moreover, it is crucial to evaluate the monetary and health-related effectiveness of specific health programs, interventions, and measures in terms of their impact on public health outcomes and thus budgets scaling vs. factual health of the public in being able to rate their return on investment, considering the different settings of nations' health care systems.

The presented consensus statements, though perhaps not immediately implementable worldwide, can serve as a crucial step toward establishing a roadmap for best practices in health science. To facilitate practical application, certain initial entry points can help guide implementation. Promoting “Healthy Eating & Active Living” (through well-planned wholefood, plant-predominant diets and regular PA) provides a foundational approach to prevent NCDs and improve overall health and well-being. Early interventions targeting children and adolescents, alongside personalized health behavior programs for adults, can foster sustainable lifestyle habits. In parallel, enhancing health education and literacy across all educational levels equips individuals with the knowledge, skills, and motivation to adopt and maintain healthier behaviors throughout life. Together, these strategies offer a pragmatic starting framework for translating the broader 101 consensus statements into actionable measures and to ensure optimal vertical permeability at all relevant levels and settings. This approach aims to address today's public health challenges, particularly in the increasing prevalence of NCDs and their underlying mechanisms, that are often neglected and/or forgotten, yet often need to be addressed despite growing advance in health science and increasing healthcare budgets. By providing practical strategies and highlighting research priorities and policies, the present consensus report and its detailed statements can assist in developing up-to-date and effective approaches that emphasize de-medicalized, holistic, and integrated health perspectives. They hold the potential to bridge existing gaps and limitations in health-related approaches and ensure that future efforts are aligned with the latest scientific findings.

The present consensus statements can contribute to the development of effective approaches, aiming for the overall improvement of individual and public health. This is especially relevant for policy and decision-makers, as well as various stakeholders, including NGOs, globally acting organizations, research funding agencies, health experts, scientists, healthcare and educational professionals, educational entities and institutions, and health-related industries.

## Author disclaimer

The views, opinions, and findings expressed within this multidisciplinary consensus statement paper and contained in this report are those of the authors and should not be construed or interpreted as representing the official viewpoint, guideline or decision of the Department of Research and Development in Teacher Education of the University College of Teacher Education Tyrol, Innsbruck, Austria, or representing exhaustingly the state-of-the-art science from the cross-cut area at the interface of or overlapping/inter-disciplinary areas medicine and health sciences, life sciences, sport science, and nutrition science, respectively. Approved for public release; distribution unlimited.
